# Totum‐448 Improves MASLD and Modulates Microbiota in Hamsters: Dose–Response Study and Effects of Supplementation Cessation

**DOI:** 10.1002/fsn3.70904

**Published:** 2025-09-02

**Authors:** Vivien Chavanelle, Marie Vallier, Yolanda F. Otero, Doriane Ripoche, Florian Le Joubioux, Thierry Maugard, Gaël Ennequin, Valérie Hervieu, Sébastien Peltier, Pascal Sirvent

**Affiliations:** ^1^ Valbiotis R&D Center Valbiotis Périgny France; ^2^ Equipe BCBS (Biotechnologies et Chimie des Bioressources pour la Santé), UMR CNRS 7266 LIENSs La Rochelle Université La Rochelle France; ^3^ Laboratoire des Adaptations Métaboliques à l'Exercice en conditions Physiologiques et Pathologiques (AME2P), UR 3533 Université Clermont Auvergne Clermont‐Ferrand France; ^4^ Department of Pathology Hospices Civils de Lyon Lyon France; ^5^ Université de Lyon Lyon France

**Keywords:** fibrosis, inflammation, liver, microbiota, nutraceuticals, preclinical trial, steatosis

## Abstract

Metabolic dysfunction‐associated steatotic liver disease (MASLD) is a global condition linked to obesity. Totum‐448, a polyphenol‐rich blend of five plant extracts and choline, was developed to target MASLD progression. This study evaluated the dose‐dependent effects of Totum‐448 and their sustainability after supplementation cessation on MASLD features and cecal microbiota. Male hamsters were fed a normal diet, a Western diet (WD), or WD supplemented with Totum‐448 (3.5% or 5% w/w) for 12 weeks (dose–response study). A parallel 18‐week on/off study included 12 weeks of Totum‐448 (5% w/w) followed by a 6‐week cessation period. Totum‐448 dose‐dependently reduced hepatic and circulating triglycerides, total cholesterol, and free fatty acids, independently of any changes in body composition. Gene markers of liver inflammation (*Tgfb1*, *Il1b, Ccl2*, *Il6*) and fibrosis (*Col3a1, Vcam*) were downregulated with the highest dose of Totum‐448 only. Supplementation cessation led to a gradual rebound in serum and liver lipid levels and in relative expression of gene markers of hepatic inflammation and fibrosis, resulting in the loss of the majority of the benefits conferred by Totum‐448, although partial effects on hepatic steatosis and hepatocyte ballooning were maintained. Cecal microbiota analysis revealed modulation of the relative abundance of *Acetatifactor*, *Alloprevotella*, *Lactobacillus*, and *Lawsonibacter*, with identified correlations with certain metabolic outcomes. In conclusion, Totum‐448 demonstrated dose‐dependent improvements in key MASLD features in WD‐fed hamsters which gradually diminished after supplementation stopped. These findings underscore its therapeutic potential in MASLD management. The correlations with microbiota changes suggest a possible gut–liver axis role in its effects.

## Graphical abstract

Totum‐448 is a blend of 5 plant extracts and choline designed to tackle early stages of MASLD. This work is the first study to evaluate the effect of Totum‐448 in a hamster model of the pathology. Totum‐448 demonstrated dose‐dependent improvements in key MASLD features in WD‐fed hamsters which gradually diminished after supplementation stopped. The correlations with microbiota changes suggest a possible gut‐liver axis role in its effect.

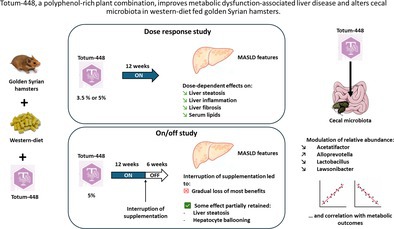

## 1 Introduction

Metabolic dysfunction‐associated steatotic liver disease, MASLD, formerly known as nonalcoholic fatty liver disease (NAFLD) (Rinella, Lazarus, et al. 2023) has become the most frequent liver pathology in the world, with an estimated global prevalence of 30% to 32% forecasted to increase as the obesity pandemic develops (Riazi et al. 2022; Younossi et al. 2023). It is a progressive disease that begins with simple lipid infiltration in the hepatocytes (steatotic liver disease) before advancing to MASH (metabolic dysfunction‐associated steatohepatitis), formerly known as NASH (nonalcoholic steatohepatitis), as inflammation and hepatocyte ballooning develop. This chronic inflammatory state can lead to fibrosis, where scar tissue replaces healthy liver tissue, ultimately worsening to life‐threatening stages of cirrhosis and hepatic decompensation, potentially requiring liver transplant (Allen et al. 2022). To date, only one molecule (*Resmetirom*, a thyroid hormone receptor beta agonist) was approved by the *Food and Drug Administration* for the treatment of MASLD (FDA 2024) in the United States of America. While well tolerated, this molecule lacks long‐term follow‐up data, and undesirable side effects including nausea and diarrhea were reported (Dutta et al. 2024). Additionally, its cost is anticipated to be elevated (Javanbakht et al. 2022), potentially restricting its broader applicability.

Since MASLD is a largely preventable disease, major health organizations worldwide recommend lifestyle modifications, such as dietary adjustments and increased physical activity, as first‐line interventions (Rinella, Neuschwander‐Tetri, et al. 2023; Tacke et al. 2024). These approaches demonstrate significant short‐term benefits but suffer from poor long‐term adherence in many patients. In this framework, polyphenols have emerged as a promising therapeutic option due to their well‐documented effects on the hallmarks of MASLD pathophysiology, such as steatosis, inflammation, or fibrosis (Ranneh et al. 2024), with certain molecules having shown promising results in clinical trials (Huang et al. 2023). Recently, growing interest has also been directed toward the role of the gut microbiota in the pathogenesis and management of MASLD (Maher et al. 2024). Alterations in gut microbiota composition, also referred to as dysbiosis, have been linked to key features of MASLD, including liver fat accumulation (Hullar et al. 2021), fibrosis (Zazueta et al. 2024), or more generally, MASLD severity (Manzoor et al. 2022). Hence, modulating the gut‐liver axis via microbiota‐targeted interventions could present innovative therapeutic opportunities. Polyphenols, through their mode of administration (*per os*), appear as good candidates for microbiota modulation, and incidentally, previous works have shown that part of their anti‐MASLD action could be exerted through modulation of microbiota (Mohammadhasani et al. 2024).

In this framework, Totum‐448 was developed as a potential intervention to prevent the progression of early‐stage MASLD to MASH. Totum‐448 is a patented blend of extracts obtained from olive leaf ( *Olea europaea* ), bilberry ( *Vaccinium myrtillus* ), artichoke leaf ( *Cynara scolymus* ), chrysanthellum (*Chrysanthellum indicum subsp. afroamericanum B.L. Turner*), black pepper ( *Piper nigrum* ), and choline. Each ingredient was included for its complementary bioactive properties targeting metabolic and liver health (Patent FR1460064A), based on an initial scan of the existing literature and evaluation of regulatory constraints, followed by subsequent in‐house individual and combination tests (unpublished conditional industrial data). This study represents an initial exploration of the potential of Totum‐448 as a therapeutic approach, aiming to provide foundational insights into its effects on MASLD and its progression. Given the complexity of MASLD and its resistance to treatment, relatively high doses of the supplement were chosen for this preclinical study to maximize the potential for observing meaningful effects. This trial was conducted in western diet (WD)‐fed hamsters, a well‐established model of diet‐induced MASLD (Briand et al. 2018, 2021, 2012; Gao et al. 2010; Lai et al. 2016; Svop Jensen et al. 2021; Tréguier et al. 2011), closely recapitulating several human MASLD features, with two primary objectives:

- to assess the dose‐dependent effects of Totum‐448 on MASLD progression, and
- to evaluate the sustainability of Totum‐448's effects after a 12‐week supplementation period, followed by a 6‐week interruption of the treatment.

We hypothesized that Totum‐448 would exert dose‐dependent beneficial effects on MASLD‐related features, with these effects gradually waning after supplementation cessation, yet remaining partially preserved. MASLD‐related features were assessed in WD‐fed hamsters to test these hypotheses. Because of the possible interactions between polyphenols and microbiota, we also analyzed the modulations in microbiota induced by the different interventions and looked for possible correlations with changes in MASLD‐related outcomes.

## 2 Methods and Materials

### 2.1 Animals

All the animal procedures were approved by the local ethics committee (C2E2A, Auvergne, France, under the number #32212‐2021063014575266 accepted on October 22nd, 2021), and comply with ARRIVE guidelines, as well as the EU Directive 2010/63 for the protection of animals used for scientific purposes. Six‐week‐old male golden Syrian hamsters were provided by Janvier Labs (Le Genest‐Saint‐Isle, France), with a specific pathogen‐free health status. All the hamsters were housed at 22°C under a standard 12 h light–12 h dark cycle, 3 hamsters per cage. Upon arrival, hamsters were acclimatized for 2 weeks and fed a purified normal diet (ND, Table 1). Animals had access to food and water *ad libitum*. The number of animals per group was determined based on previous studies in hamsters (Langhi et al. 2023) and was reduced to 6 in group ND to minimize the total number of animals used, in accordance with the 3Rs (Russell and Burch 1959), because comparisons to that group did not constitute an objective in this work. Animals were monitored daily for morbidity, mortality, or adverse clinical signs. Animals were randomly assigned to the experimental groups based on body weight and fat mass using Randomice software v.1.17 (van Eenige et al. 2020). The purified diets were manufactured by Research Diets (NJ, USA, their composition is available in Table 1). The relative amounts of essential nutrients (cellulose, vitamins, …) in both diets were matched by their total calorie content, not by weight (Stevenson and Ward 2022). Totum‐448 was provided by Valbiotis (Perigny, France) and incorporated into the WD at 3.5% or 5% w/w by Research Diets. Its chemical characterization is available (Table S1).

**TABLE 1:** Composition of experimental diets of hamsters.

Macronutrient	ND (D16010603)	WD (D99122211)
	% kcal	% kcal
Protein	20	20
Carbohydrate	70	35
Fat	10	45
kcal/g	3.85	4.66

Ingredient	g	kcal	g	kcal
Casein	200.00	800	200.00	800
l‐Cystine	3.00	12	3.00	12
Corn starch	452.20	1809	72.80	291
Maltodextrin 10	75.00	300	100.00	400
Fructose	0.00	0	172.80	691
Sucrose	172.80	691	0.00	0
Cellulose	50.00	0	50.00	0
Soybean oil	25.00	225	25.00	225
Coconut oil, hydrogenated	20.00	180	177.50	1598
Mineral mix S10026	10.00	0	10.00	0
Dicalcium phosphate	13.00	0	13.00	0
Calcium carbonate	5.50	0	5.50	0
Potassium citrate	16.50	0	16.50	0
Vitamin mix V10001	10.00	40	10.00	40
Choline bitartrate	2.00	0	2.00	0
Cholesterol	0.00	0	12.50	0
Dye	0.00	0	0.05	0
Total	1055.00	4057	870.65	4057

Abbreviations: ND, normal diet; WD, western diet.

#### 2.1.1 Dose–Response Study

Animals were assigned to the following groups: ND (*n* = 6), western diet (WD, *n* = 12), WD+Totum‐448 3.5% w/w (WD‐T448 3.5%, *n* = 12) and WD+Totum‐448 5% w/w (WD‐T448 5%, *n* = 12). The average body weight of the animals before group attribution was 89 g (ranging from 76 to 106 g). The study duration was 12 weeks.

#### 2.1.2 On–Off Study

Animals were randomly assigned to experimental groups: ND (*n* = 6), WD (*n* = 12), WD+Totum‐448 5% w/w (WD‐T448, *n* = 12), and WD‐T448 ON/OFF (12 weeks of WD‐T448, “ON,” followed by 6 weeks of WD, “OFF,” *n* = 12). The average body weight of the animals before group attribution was 95 g (ranging from 74 to 109 g). The study duration was 18 weeks.

### 2.2 Body Parameters and Food Intake

Body weight was recorded once weekly. Body composition (fat mass and lean mass) was determined by magnetic resonance imaging (Echo MRI, Zynsser Analytic, Fürstenfeldbruck, Germany). Food intake was measured three times weekly and averaged by cage.

### 2.3 Serum Parameters

#### 2.3.1 Glycemia, Insulin, Total Cholesterol (TC), and Triglycerides (TG)

Blood was collected from the gum vein of 5–6‐h fasted animals, and fasting glycemia was measured from a drop of fresh whole blood with a StatStrip Glucose Xpress (Nova Biomedical Corp., Waltham CA, USA). The rest of the whole blood was left to clot at room temperature for 30 min before being centrifuged for 10 min at 2000 *g*. Serum (supernatant) was collected and stored at −80°C until analysis. Fasting serum insulin was assessed using a hamster Insulin ELISA Kit provided by Crystal Chem (#90336, Elk Grove Village IL, USA) following the instructions of the manufacturer. Absorbance was read at 450 nm in a multiplate reader (Spark 10M, Tecan, CH). Serum TC levels were assessed using an enzymatic assay kit provided by Biolabo (#LP80106, Maizy, France). Serum samples were diluted 10 times in NaCl 0.9%, and absorbance was read in a multiplate reader (Spark 10M, Tecan, CH). Serum TG levels were assessed using a TG enzymatic assay kit provided by Cayman Chem (#10010303, Ann Arbor, MI, USA). Serum samples were diluted 10 times in sodium phosphate assay buffer; absorbance was read at 540 nm in a multiplate reader (Spark 10M, Tecan, CH). For all colorimetric measurements, samples whose technical duplicates did not fall within 20% of their coefficient of variation were removed.

#### 2.3.2 Free Fatty Acids (FFA), Aspartate Aminotransferase (AST) and Alanine Aminotransferase (ALT)

Blood was collected by cardiac puncture in 5–6 h fasted anesthetized animals before euthanasia, and serum was prepared and stored as described hereabove. Serum free fatty acids were assessed using a Free Fatty Acid quantification kit provided by Sigma‐Aldrich (#MAK044, Merck, St Louis MO, USA) following the manufacturer instructions. Absorbance was read at 570 nm in a multiplate reader (Spark 10M, Tecan, CH). Serum AST activity was measured with an aspartate aminotransferase activity assay kit provided by Abcam (#Ab105135, Cambridge UK). AST activity was assessed following the manufacturer instructions based on the detection of glutamate produced by enzymatic reaction at 450 nm for 60 min in a multiplate reader (Tecan, CH), plotted against a glutamate standard curve (Magellan Spark Control, Tecan, CH). Serum ALT activity was estimated using an alanine transaminase activity assay kit provided by Abcam (#Ab105134, Cambridge UK). ALT activity was assessed following the manufacturer instructions based on the detection of pyruvate produced by enzymatic reaction at 570 nm for 60 min in a multiplate reader (Spark 10M, Tecan, CH), plotted against a pyruvate standard curve (Magellan Spark Control, Tecan, CH).

### 2.4 Tissue Collection

Fasted hamsters (5–6 h) were anesthetized with isoflurane and euthanized by cervical dislocation. Liver and cecal content were carefully dissected and processed as described thereafter. Additionally, for the on–off experiment, adipose tissue pads were sampled and weighed.

### 2.5 Liver Lipids

Livers (10–100 mg) were homogenized in 1 mL of a solution containing 5% NP40 in water using a rotor homogenizer (Ultra‐Turrax T10, IKA, Germany) for 20 s at maximum speed (30,000 rpm). Homogenization was visually assessed, and the process was repeated if any tissue fragments remained. Homogenates were heated to 80°C–100°C for 2–5 min before being cooled to room temperature. The whole process was repeated once; then, samples were centrifugated (2000 *g*—2 min). Liver TG content was determined using an enzymatic assay kit provided by Cayman Chem (#10010303, Ann Arbor, MI, USA) following the instructions, as described above. Liver TC level was assessed using an enzymatic assay kit provided by Biolabo (#LP80106, Maizy, France) following the manual, as described above. Liver FFA were assessed using a Free Fatty Acid quantification kit provided by Sigma‐Aldrich (#MAK044, Merck, St Louis MO, USA). For all colorimetric dosages in liver homogenate, samples whose technical duplicates did not fall within 20% of their coefficient of variation were removed.

### 2.6 Liver Histology

Livers were fixed for 48 h at 4°C in formalin (4% paraformaldehyde) and kept in ethanol 70% at 4°C for at least 3 h (up to overnight) before automated tissue processing (Histocore Pearl, Leica Biosystems, Germany). They were then embedded in paraffin, and sections were cut at 4 μm with a microtome before being stained with Hematoxylin and Eosin (H&E) or Sirius Red.

#### 2.6.1 H&E Staining

Sections were heated at 56°C for 15 min and dewaxed in xylene (3 baths: 10 min, then twice 5 min) then rehydrated in four successive ethanol baths (100%, 95%, 80%, and 70%) for 3 min each, followed by 1 immersion in ultra‐pure water (3 min). Thereafter, sections were stained in a bath composed of Gill II hematoxylin (#GHS232 Sigma‐Aldrich) for 4 min, washed twice in water for 10 min each before being immersed in 0.25% eosin Y (#HT110116, Sigma‐Aldrich) for 45 s. Finally, sections were dehydrated with ethanol (3 baths: 95%, 100%, and 100%, for 3 min each) and xylene (3 min), and slides were mounted with Eukitt medium (#03989 Sigma‐Aldrich).

#### 2.6.2 Sirius Red Staining

Sections were dewaxed in xylene then rehydrated in four successive ethanol baths (100%, 95%, 80%, and 70%) for 10 s each. Thereafter, they were stained in Sirius red (#365548, Sigma‐Aldrich) 0.1% in picric acid for 1 h, and, after removal of excess stain in acetic acid (0.5% in distilled water), sections were dehydrated with 4 ethanol baths (70%, 80%, 90%, and 100%) for 10 s followed by 2 xylene immersions (3 min each). Slides were then mounted with Eukitt medium.

Microtome blades were analyzed in blinded conditions for scoring of inflammation, ballooning, and fibrosis, as described previously (Lavrut et al. 2023). Inflammation was graded from 0 (no inflammation) to 3 (significant inflammation); fibrosis of the liver parenchyma was evaluated using a score corresponding to 0 = none, 1 = mild (portal or pericellular fibrosis), 2 = moderate (thin and diffuse or thick but occasional fibrosis) and 3 = thick and diffuse fibrosis. The ballooning extent was assessed as a percentage of ballooned cells.

### 2.7 Gene Expression in the Liver

Total mRNA was extracted from liver and ileum using TRIzol (Invitrogen, Life Technologies, CA, USA) following the manufacturer's instructions. cDNA was synthesized from 2 μg RNA with the High‐Capacity cDNA transcription kit (Applied Biosystems, Life Technologies, CA, USA) in a T100 Thermal Cycler (Bio‐Rad, CA, USA) for 10 min at 25°C, 120 min at 37°C, and 5 min at 85°C, in the presence of a RNase inhibitor (Ambion 0.2 U/μL, # AM2682, Thermo Fisher Scientific, MA USA). Real‐time quantitative PCR amplification was carried out using the CFX system (Bio‐Rad, USA) with Sybr Green probes (Eurofins, Luxemburg) and Sybr Green Master Mix (SYBR Select Master Mix, Thermo Fischer Scientific, USA). The relative expression of target genes was calculated with the ΔΔCt method using *Polr2a* (dose–response study) or *Tuba1c* (on/off study) as housekeeping genes and the respective ND group as a reference.

Data are expressed as relative quantity using the following formula:

$$\mathrm{Rq}=2{-}^{∆∆\mathrm{Ct}}$$

where Δ*C*_*t*_ = *C*_*t*_ (target) − *C*_*t*_ (housekeeping gene) and ΔΔ*C*_*t*_ = Δ*C*_*t*_ (sample) − Δ*C*_*t*_ (control).

Sybr green probes used in this work are available (Table 2).

**TABLE 2:** Sequences of SYBR green probes used for qPCR assays in the liver of hamsters.

Gene	Forward sequence	Reverse sequence
*Ccl2*	TGCTAACTTGACGCAAGCTCC	AAGTTCTTGAGTCTGCGGTGG
*Il1b*	GCAACTGTTCCTGAACTCGAC	TGGATAGCTCAGGTCAAGGCT
*Tgfb1*	ATTCGGGAAGCAGTGCCTGA	ACGCCAGGTATTGTTGCTGT
*Il6*	CCTGGAGTTTGTGACGAACAAT	GTTGGGCTAGGCGTGACTATT
*F4/80*	GCTTCCAACCAGAGCCAGAA	CCTGCTTGGCACTGCTGTAT
*Col1a1*	CTGACGCATGGCCAAGAAGA	CGTGCCATTGTGGCAAATACA
*Col3a1*	GGCTCTCCTGGAATCTGTACA	GGATAGCCACCAATTCCTCCT
*Col6a1*	AAAGGCACCTACACCGACTG	TCGGTCACCACGATCAGGTA
*Acta2*	GATCCTGACTGAACGTGGCT	CCAGCCGACTCCATCCCAAT
*Vcam*	ACTGCAAGTCTACACATCTCCC	GGTAGACCCTCACTGGAGCA
*Tuba1c*	TCCTTGGTAGTCTACTAGTGGGAG	TGGAGATGCACTCACGCATGTT
*Polr2a*	AGGAACACATGCCACCTTTCA	GAAATGGGAATGTCACACAGCA

### 2.8 Analysis of Cecal Microbiota

DNA was extracted from 50 to 100 mg of frozen cecal content using a FastDNA Spin Kit for Feces (#116570200, MP Biomedical, CA, USA) and a FastPrep‐24 5G (#116005500, MP Biomedical, CA, USA) following the manufacturer instructions. DNA was eluted in 50 μL of elution buffer, and concentration was normalized at 20 ng/μL.

Microbial 16S library preparation was performed at the PGTB (Plateforme Génome Transcriptome de Bordeaux, France) by amplification and sequencing of the V3‐V4 region of the 16S rRNA gene on an Illumina MiSeq machine, using the 2 × 250 bp Illumina v2 kit. Quality filtering and processing was done as described previously (Vallier et al. 2023) using cutadapt v4.0 (Martin 2011) with Python v3.9.9, usearch v11.0.667 (Edgar 2016; Edgar and Flyvbjerg 2015) and mothur v1.48.0 (Schloss et al. 2009). Classification of quality filtered reads was performed by comparison with the ribosomal database project (RDP trainset 19) (Cole et al. 2014; Quast et al. 2012).

### 2.9 Statistical Analyses

#### 2.9.1 General

Values are presented as mean ± SD. Differences were considered statistically significant at *p* < 0.05. Statistical analysis was performed using GraphPad Prism V.10.3.0. For comparisons of means between groups, a Shapiro–Wilk normality test was used to determine whether the data were consistent with a Gaussian distribution. If data were not distributed according to the normal distribution, a Kruskal–Wallis nonparametric test was used followed by Dunn's test for post hoc comparisons. When normal distribution was assumed, measures were subjected to a one‐way ANOVA or Welch‐corrected one‐way ANOVA (if conditions of equivalence of variances were not met assessed by a Fisher *F*‐test) followed by Šídák's or Dunnett's test for multiple comparisons, respectively, when ANOVA reached the significance threshold. When comparing variables measured over time, a repeated‐measures two‐way ANOVA was performed followed by Šídák's post hoc test for multiple comparisons. If a sample was missing, making it impossible to run a two‐way ANOVA, a mixed‐effects model followed by Dunnett's test for multiple comparisons was used instead. For categorical variables, pairwise comparisons were performed using a Chi‐square test. All post hoc comparisons were run without group ND, presented in the graphs for reference only.

#### 2.9.2 Microbiota

Abundance tables were imported and analyzed in R v4.1. Alpha and beta diversity were estimated using package vegan 2.6‐2 (Dixon 2003) and tested using linear models and permutational MANOVA (Adonis) tests respectively, as described previously (Belheouane et al. 2020; Vallier et al. 2023). Indicator taxa analysis was performed using linear models, as described previously (Belheouane et al. 2020; Vallier et al. 2023). A taxon was designated as an indicator if the group parameter and the overall model were statistically significant (*p* < 0.05). Correlations between indicator bacterial taxa and biological parameters were assessed through linear models in which the relative abundance of the bacterial taxon was used as the response variable, while the experimental group biological parameter and their interaction were used as explanatory variables (Belheouane et al. 2020; Vallier et al. 2023). The square sum of variances explained by the model, divided by the total variance, is reported in the figures as *r* ^2^ (in %). A negative sign was added to indicate a negative correlation. Here again, all statistical analyses were performed without group ND, presented in the graphs for reference only.

## 3 Results

### 3.1 Dose–Response Study (12 Weeks)

#### 3.1.1 In Vivo Measurements

**FIGURE 1:**
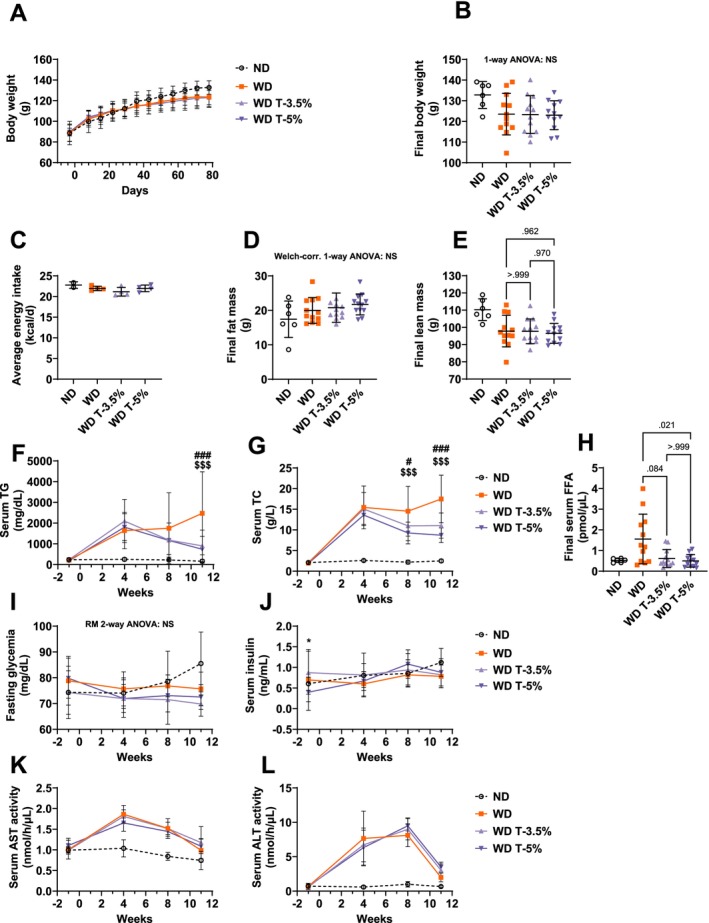
In vivo and serum parameters in the dose–response study: Totum‐448 reduced serum lipids levels without affecting energy intake, body weight, body composition, or glucose homeostasis, in WD‐fed hamsters. (A) Body weight measured weekly. For the sake of readability of the figure, statistic tests were run on final body weight only (B). (B) Final body weight (Week 12). (C) Energy intake in each cage, averaged per animal. *N* = 2–4 cages (3 animals per cage). Because of the low number of replicates, no statistical tests were carried out on energy intake. (D) Final fat mass (Week 12). (E) Final lean mass (Week 12). Serum TG (F), serum TC (G), whole blood glycemia (I), serum insulin (J), serum AST activity (K), and serum ALT activity (L) measured at Week ‐1, Week 4, Week 8, and Week 11 in blood collected in vivo from the gum vein of the animals. (H) Final serum FFA (Week 12) measured in blood collected from cardiac puncture in anesthetized animals. *N* = 6–12 animals. Post hoc comparisons are displayed on the graph if ANOVA (one‐way or repeated‐measures two‐way, or nonparametric equivalent) was significant (*p* < 0.05). WD T‐3.5% vs. WD T‐5%: *p < 0.05. WD vs. WD T‐3.5%: ^#^ *p* < 0.05, ^###^ *p* < 0.001. WD vs. WD T‐5%: ^$$$^ *p* < 0.001. In graphs K and L, mixed model interaction was < 0.05 but no post hoc comparisons were significant. ALT, alanine transaminase; AST, aspartate transaminase; FFA, free fatty acids; NS, not significant; TC, total cholesterol; TG, triglycerides.

Although no statistical tests were run between group ND and the other groups, body weight appeared to be reduced in WD group at the end of the study (123.5 ± 10.0 vs. 132.8 ± 6.5 g, Figure 1A,B), with decreased lean mass (97.8 ± 9.2 vs. 110.3 ± 6.3 g, Figure 1C) and increased fat mass (20.0 ± 3.7 vs. 17.4 ± 5.3 g, Figure 1D). Despite the higher energy density of WD, average energy intake was not increased in group WD, compared to ND (22.0 ± 0.5 vs. 22.8 ± 0.8 kcal/days, Figure 1C), suggesting that the animals may have adapted their intake to the caloric density of the diet. At the end of the study, no significant differences were noted between WD and Totum‐448 supplemented animals in body weight (WD: 123.5 ± 10.0, WD T‐3.5%: 123.3 ± 9.2, and WD T‐5%: 123.0 ± 7.0 g, one‐way ANOVA nonsignificant, Figure 1B) or body composition (For lean mass, WD vs. WD T‐3.5%: 97.8 ± 9.2 vs. 97.7 ± 7.2 g, *p* > 0.999, WD vs. WD T‐5%: 97.8 ± 9.2 vs. 96.5 ± 5.7 g, *p* = 0.962, Figure 1D. For fat mass, WD: 20.0 ± 3.7, WD T‐3.5%: 20.8 ± 4.3, WD T‐5%: 21.7 ± 3.0 g, one‐way ANOVA nonsignificant, Figure 1E). In the serum, TG appeared to be elevated in all 3three WD groups from Week 4 and on, compared to ND. Totum‐448 supplementation in groups WD T‐3.5% and WD T‐5% significantly decreased serum TG at Week 11, compared to WD (900.9 ± 220.8 and 740.3 ± 179.5 vs. 2473.3 ± 578.3 mg/dL, *p* < 0.001 and *p* < 0.001, respectively, Figure 1F). No statistical difference was observed between the two Totum‐448 doses for serum TG. Similarly, serum TC was elevated from Week 4 and on in all WD groups, compared to ND. Totum‐448‐supplemented animals in groups WD T‐3.5% and WD T‐5% displayed reduced serum TC at Week 8, vs. WD (11.0 ± 1.0 and 9.3 ± 0.8 vs. 14.5 ± 1.7 g/L, *p* < 0.05 and *p* < 0.001, respectively, Figure 1F) and at Week 11 (11.1 ± 0.9 and 8.7 ± 0.5 vs. 17.5 ± 1.7 g/L, *p* < 0.001 and *p* < 0.001, respectively, Figure 1G). No statistical differences were noted between the two Totum‐448 doses for serum TC. No differences between groups were observed in fasting glycemia (Two‐way repeated‐measures ANOVA interaction, *p* = 0.075, Figure 1I) or in fasting insulinemia (except before the start of the study, at Week ‐1 between WD T‐3.5% and WD T‐5%, Figure 1J). Finally, serum transaminases were also elevated in all three WD groups, compared to ND, but we did not observe any significant effect of Totum‐448 (Figure 1K,L).

#### 3.1.2 Serum FFA

Serum FFA assessed at the end of the study in blood collected from cardiac puncture in anesthetized animals was significantly reduced in WD T‐5% vs. WD (0.50 ± 0.30 vs. 1.56 ± 1.20 pmol/μL, *p* = 0.021, Figure 1H), while the difference between WD T‐3.5% and WD was not significant (*p* = 0.084).

#### 3.1.3 Liver Parameters

**FIGURE 2:**
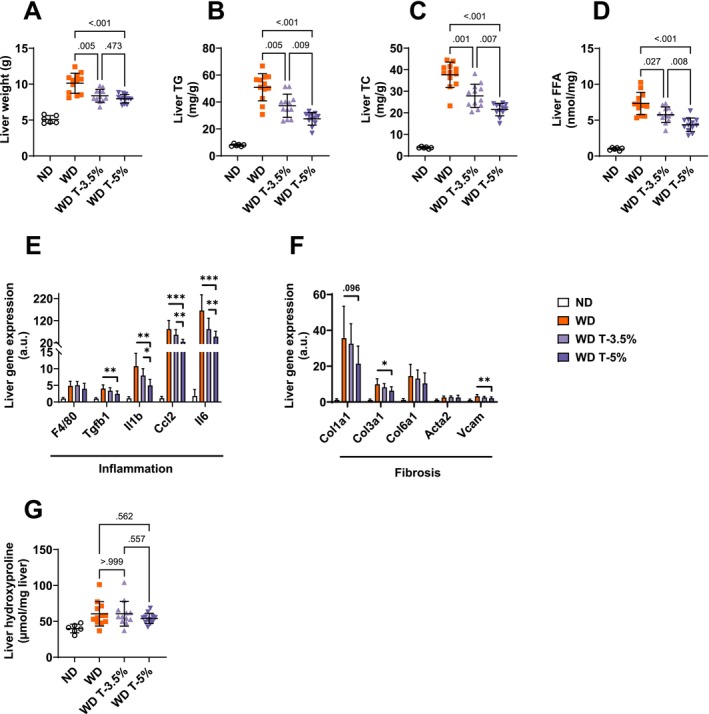
Liver markers in the dose–response study: Totum‐448 dose‐dependently reduced liver lipids and gene markers of inflammation and fibrosis, in WD‐fed hamsters. (A) Liver weight. (B) Liver TG, in mg per gram of wet tissue. (C) Liver TC, in mg per gram of wet tissue. (D) Liver FFA, in nmol per milligram of wet tissue. (E) Relative expression of genes associated with inflammation. (F) Relative expression of genes associated with fibrosis. (G) Liver hydroxyproline expressed in μmol per milligram of wet tissue. *N* =6 to 122 animals. Post hoc comparisons are displayed on the graph if ANOVA (one‐way or nonparametric equivalent) was significant (*p* < 0.05). *p < 0.05, **p < 0.01, ***p < 0.001. FFA, free fatty acids. TC, total cholesterol; TG, triglycerides.

**FIGURE 3:**
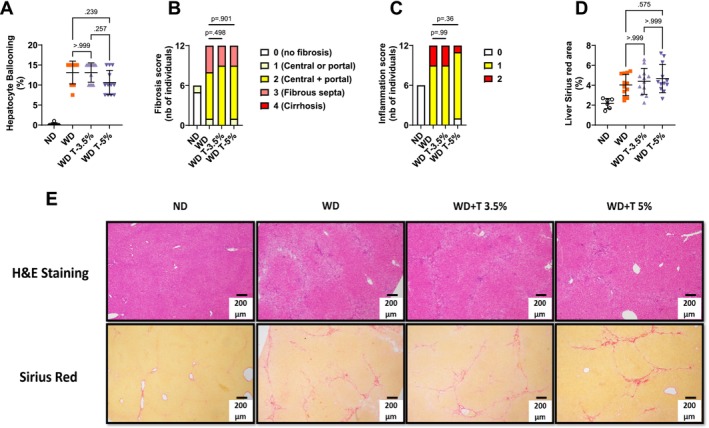
Liver histology in the dose–response study: No effect of Totum‐448 was observed in hepatocyte ballooning, fibrosis, or inflammation, as assessed by histological scoring and Sirius red‐dyed area, in WD‐fed hamsters. (A) Hepatocyte ballooning. (B) Fibrosis scoring. (C) Inflammation scoring. (D) Sirius red‐dyed area. (E) Representative images of H&E and Sirius red‐stained liver sections. *N* = 6–12 animals. (A, D) Post hoc comparisons are displayed on the graph if ANOVA (one‐way or nonparametric equivalent) was significant (*p* < 0.05). (B, C) *p* values indicate pairwise *χ* ^2^ tests. H&E, hematoxylin and eosin.

WD‐induced increase in liver weight was partially prevented in groups WD T‐3.5% and WD T‐5% versus WD (8.38 ± 0.26 and 7.97 ± 0.17 vs. 10.13 ± 0.40 g, *p* = 0.005 and *p* < 0.001, respectively, Figure 2A). All liver lipids were elevated in group WD compared to ND (TG, TC and FFA, Figure 2B–D, respectively). Totum‐448 supplementation resulted in a statistically significant dose‐dependent reduction thereof. Specifically, liver TG content was significantly reduced in WD T‐3.5% versus WD (37.24 ± 2.49 vs. 50.92 ± 2.92 mg/g liver tissue, *p* = 0.005, Figure 2B) and to a greater extent in WD T‐5%, compared to WD T‐3.5% (27.52 ± 1.36 vs. 37.24 ± 2.49 mg/g liver tissue, *p* = 0.009). Liver TC content was significantly reduced in WD T‐3.5% versus WD (27.86 ± 1.57 vs. 37.63 ± 1.70 mg/g liver tissue, *p* = 0.001, Figure 2C) and to a greater extent in WD T‐5%, compared to WD T‐3.5% (21.52 ± 0.84 vs. 27.86 ± 1.57 mg/g liver tissue, *p* = 0.007). Liver FFA content was significantly reduced in WD T‐3.5% vs. WD (5.76 ± 0.31 vs. 7.33 ± 0.45 nmol/mg liver tissue, *p* = 0.027, Figure 2D) and to a greater extent in WD T‐5%, compared to WD T‐3.5% (4.35 ± 0.27 vs. 5.76 ± 0.31 mg/g liver tissue, *p* = 0.008). The relative expression of gene markers of inflammation was assessed in the liver by qPCR. We found elevated *F4/80*, *Tgf1b*, *Il1b*, and *Ccl2* expression in group WD. Totum‐448 at the highest dose only (WD T‐5%, vs. WD) significantly blunted the elevation of *Tgfb1* (0.6‐fold decrease, *p* < 0.01), *Il1b* (0.5‐fold decrease, *p* < 0.01), *Ccl2* (0.3‐fold decrease, *p* < 0.001), and *Il6* (0.3‐fold decrease, *p* < 0.001, Figure 2E). Similarly, WD exposure resulted in elevated relative gene expression of markers of fibrosis (*Col1a1*, *Col3a1*, *Col6a1*, *Acta2*, and *Vcam*, Figure 2F). This increase was partially prevented only with the highest dose of Totum‐448 (WD T‐5% vs. WD) for *Col1a1* (0.6‐fold decrease, trend only, *p* = 0.096), *Col3a1* (0.6‐fold decrease, *p* < 0.05), and *Vcam* (0.7‐fold decrease, *p* < 0.01). Additionally, elevated hydroxyproline levels (a marker of collagen content) were found in all WD groups, compared to ND, without any significant effect of Totum‐448 supplementation (Figure 2G). Finally, histological analyses of liver revealed no significant effect of supplementation in hepatocyte ballooning assessment (Figure 3A), fibrosis (Figure 3B), or inflammation score (Figure 3C). In line with this, no differences were observed between groups in Sirius Red‐dyed area (Figure 3D). Representative images of H&E and Sirius Red‐stained sections are shown in Figure 3E.

#### 3.1.4 Cecum Microbiota

##### 3.1.4.1 Average Relative Composition

Average relative composition at phylum, class, order, family, and genus level is presented in Figure S1 (A, B, C, D and E, respectively). Differences between ND and WD are visible. Although no statistical analysis was carried out, quite unexpectedly, a pattern seems to emerge in Totum‐448‐supplemented groups, with a tendency to exacerbate the existing differences between WD and ND (further decrease of relative abundance of a given taxon with Totum‐448 when the latter was already decreased by WD, vs. ND, and vice versa).

##### 3.1.4.2 Alpha and Beta‐Diversity

Alpha diversity was assessed by Chao and Shannon indexes at all five levels precedingly mentioned. No differences between groups were noted in the Chao index (Figure S2A–E). Significant differences among groups were found in the Shannon index at the phylum level (linear model *r* ^2^ = 0.32, *p* < 0.01, Figure S2F), with an increased Shannon index in group WD, and to a further extent in group WD T‐5%. No significant differences were found at the other levels (Figure S2G–J).

Beta diversity was assessed by Jaccard and Bray–Curtis indexes at all five levels. The Jaccard index indicated a significant group effect only at the genus level (Adonis 0.272, *p* < 0.001, Figure S3E), with group WD well separated from group ND and groups WD T‐3.5% and WD T‐5% clustering together, detached from groups WD and ND. No differences were found in Jaccard indexes at upper levels (Figure S3A–D). On the other hand, the Bray–Curtis index revealed a significant group effect at all five levels, namely: phylum level (Adonis 0.329, *p* < 0.01, Figure S3F), class level (Adonis 0.16, *p* < 0.05, Figure S3G), order level (Adonis 0.162, *p* < 0.05, Figure S3H) and genus level (Adonis 0.185, *p* < 0.001, Figure S3I). This analysis shows that the Jaccard index, which does not account for bacterial abundance, revealed a significant difference among the three WD groups. The graphical representation may indicate that the largest variance is between WD/ND on one side and WD+Totum‐448 (3.5% and 5%) on the other, suggesting that Totum‐448 supplementation induced the appearance or disappearance of certain taxa. In contrast, the Bray–Curtis index, which considers bacterial abundance, also showed a significant difference according to the statistical test. The graphical representation highlights that the primary variance is between ND and the WD groups, with a smaller divergence observed between WD and WD T‐3.5% on one side and WD T‐5% on the other, suggesting that the abundance of the major taxa is not strongly impacted by the supplementation. Taken together, these observations suggest that WD induces important changes in the prevalence and abundance of the major taxa, while Totum‐448 supplementation mainly induces changes in the prevalence of minor taxa.

##### 3.1.4.3 Indicator Taxa

**FIGURE 4:**
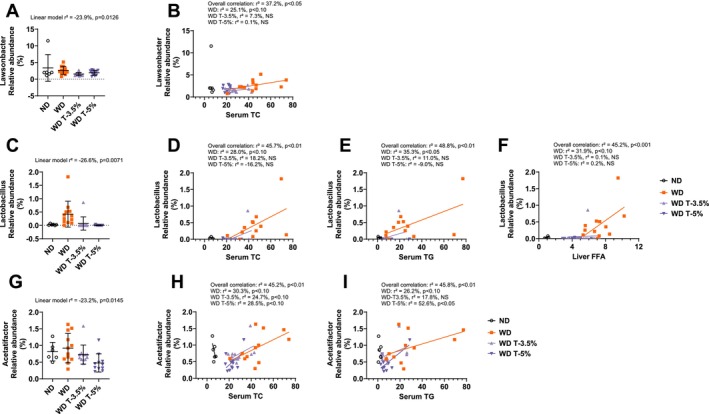
Analysis of cecal microbiota at the genus level in the dose–response study and correlations with metabolic outcomes: The relative abundance of 3 taxa at the genus level that presented a significant group effect was significantly correlated with several metabolic outcomes associated with MASLD in WD‐fed hamsters. The correlations between indicator bacterial taxa and biological parameters were assessed through linear models whenever both variables displayed a significant difference between groups. (A) *Lawsonibacter* relative abundance. (B) Significant linear correlation between *Lawsonibacter* relative abundance and serum TC. (C) *Lactobacillus* relative abundance. (D) Significant linear correlation between *Lactobacillus* relative abundance and serum TC. (E) Significant linear correlation between *Lactobacillus* relative abundance and serum TG. (F) Significant linear correlation between *Lactobacillus* relative abundance and serum FFA. (G) *Acetatifactor* relative abundance. (H) Significant linear correlation between *Acetatifactor* relative abundance and serum TC. (I) Significant linear correlation between *Acetatifactor* relative abundance and serum TG. *N* = 612 animals. Linear models. *r* ^2^ is the square sum of variances explained by the model, divided by the total variance (in %). A negative sign was added to indicate a negative correlation. FFA, free fatty acids; TC, total cholesterol; TG, triglycerides.

We performed an indicator taxa analysis at genus level. All classified genera presenting significant differences between groups are presented in Table 3 (unclassified genera with significant differences are presented in Table S2). Three distinct patterns of relative abundance were observed: a/ “Beyond WD”: Totum‐448 exacerbated the existing difference between ND and WD, be it a reduction or an increase (7 genera: *Olsenella*, *Dorea*, *Agathobaculum*, *Phocea*, *Paraprevotella*, *Alistipes*, and *Mucispirillum*), b/ “Totum‐448 specific”: No apparent difference was visible between WD and ND, but a distinction emerged in Totum‐448‐supplemented groups (12 genera: *Adlercreutzia*, *Limosilactobacillus*, *Schaedlerella, Colidextribacter*, *Dysosmobacter*, *Neglecta, Pseudoflavonifractor*, *Ruthenbacterium*, *Vescimonas*, *Duncaniella*, *Butyricimonas*, *Desulfovibrio*), and c/ “Back towards ND”: Totum‐448 appeared to counteract the effects of WD vs. ND (6 genera: *Lactobacillus*, *Lactococcus*, *Acetatifactor, Laedolimicola*, *Sporofaciens*, and *Muriventricola*). One genus (*Lawsonibacter*) was categorized as “unclear” as the group ND displayed a high variability, making it difficult to identify a directional pattern in group WD. Finally, we found that three of these indicators were also associated with a metabolic outcome. *Lawsonibacter* relative abundance decreased in Totum‐448‐supplemented groups, compared to WD (“Unclear” pattern, linear model *r* ^2^ = −0.239, *p* = 0.0126, Figure 4A), while it positively correlated with serum TC (*r* ^2^ = 0.372, *p* < 0.05, Figure 4B). Similarly, *Lactobacillus* relative abundance was reduced in Totum‐448‐supplemented groups, versus WD (“Back towards ND” pattern, linear model *r* ^2^ = −0.266, *p* = 0.0071, Figure 4C) and positively correlated with serum TC (*r* ^2^ = 0.457, *p* < 0.01, Figure 4D), serum TG (*r* ^2^ = 0.488, *p* < 0.01, Figure 4E), and liver FFA (*r* ^2^ = 0.452, *p* < 0.001, Figure 4F). Lastly, *Acetatifactor* relative abundance decreased in Totum‐448‐supplemented groups (“Totum‐448 specific” pattern, linear model *r* ^2^ = −0.232%, *p* = 0.0145, Figure 4G) and positively correlated with serum TC (*r* ^2^ = 0.452, *p* < 0.01, Figure 4H) and serum TG (*r* ^2^ = 0.458, *p* < 0.01, Figure 4I). Noteworthily, the correlation between the relative abundance and the metabolic outcome for *Lawsonibacter* and *Lactobacillus* was largely driven by the group WD.

**TABLE 3:** Analysis of cecal microbiota of hamsters in the dose–response study: Significant changes in relative abundance of classified taxa at the genus level.

Effect of T‐448	Name	Mean ± SD				*r* ^2^	*p*
		ND	WD	WD T‐3.5%	WD T‐5%		
Beyond WD	*Olsenella*	0.083% ± 0.055%	0.037% ± 0.039%	0.010% ± 0.012%	0.015% ± 0.013%	−19.16%	0.0333
	*Dorea*	0.106% ± 0.053%	0.125% ± 0.080%	0.257% ± 0.101%	0.273% ± 0.110%	33.82%	0.0014
	*Agathobaculum*	0.000% ± 0.000%	0.003% ± 0.004%	0.006% ± 0.009%	0.018% ± 0.012%	38.55%	< 0.001
	*Phocea*	0.002% ± 0.004%	0.011% ± 0.009%	0.020% ± 0.019%	0.029% ± 0.019%	17.67%	0.0445
	*Paraprevotella*	1.545% ± 0.572%	2.067% ± 1.298%	3.165% ± 2.172%	5.925% ± 2.751%	38.06%	< 0.001
	*Alistipes*	0.636% ± 0.162%	1.185% ± 0.332%	1.529% ± 0.742%	1.806% ± 0.488%	18.79%	0.0358
	*Mucispirillum*	0.089% ± 0.099%	0.440% ± 0.313%	0.437% ± 0.236%	0.790% ± 0.232%	29.49%	0.0037
T‐448 specific	*Adlercreutzia*	0.006% ± 0.004%	0.005% ± 0.009%	0.016% ± 0.011%	0.021% ± 0.016%	24.20%	0.0119
	*Limosilactobacillus*	0.059% ± 0.043%	0.115% ± 0.152%	0.001% ± 0.002%	0.017% ± 0.054%	−24.30%	0.0116
	*Schaedlerella*	0.108% ± 0.089%	0.021% ± 0.016%	0.312% ± 0.212%	0.625% ± 0.255%	64.64%	< 0.001
	*Colidextribacter*	0.014% ± 0.014%	0.011% ± 0.011%	0.012% ± 0.011%	0.002% ± 0.003%	−21.00%	0.023
	*Dysosmobacter*	0.306% ± 0.248%	0.306% ± 0.201%	0.511% ± 0.33%	0.899% ± 0.496%	33.47%	0.0015
	*Neglecta*	0.076% ± 0.085%	0.049% ± 0.032%	0.154% ± 0.034%	0.360% ± 0.173%	63.78%	< 0.001
	*Pseudoflavonifractor*	0.004% ± 0.005%	0.003% ± 0.006%	0.033% ± 0.030%	0.020% ± 0.028%	22.97%	0.0154
	*Ruthenibacterium*	0.064% ± 0.108%	0.050% ± 0.028%	0.108% ± 0.044%	0.184% ± 0.080%	52.09%	< 0.001
	*Vescimonas*	0.371% ± 0.226%	0.302% ± 0.123%	0.601% ± 0.222%	0.880% ± 0.394%	45.90%	< 0.001
	*Duncaniella*	2.189% ± 2.194%	1.757% ± 1.821%	3.945% ± 3.029%	4.196% ± 1.491%	20.97%	0.0232
	*Butyricimonas*	0.197% ± 0.074%	0.200% ± 0.062%	0.302% ± 0.100%	0.321% ± 0.103%	27.98%	0.0052
	*Desulfovibrio*	0.192% ± 0.133%	0.103% ± 0.176%	0.321% ± 0.148%	0.283% ± 0.115%	31.04%	0.0026
Back towards ND	*Lactobacillus*	0.028% ± 0.027%	0.423% ± 0.485%	0.071% ± 0.248%	0.004% ± 0.011%	−26.61%	0.0071
	*Lactococcus*	0.071% ± 0.026%	0.097% ± 0.040%	0.071% ± 0.039%	0.027% ± 0.018%	−43.78%	< 0.001
	*Acetatifactor*	0.814% ± 0.278%	0.921% ± 0.441%	0.728% ± 0.285%	0.476% ± 0.272%	−23.24%	0.0145
	*Laedolimicola*	0.617% ± 0.500%	0.256% ± 0.123%	0.388% ± 0.253%	0.513% ± 0.216%	22.08%	0.0185
	*Sporofaciens*	0.057% ± 0.065%	0.017% ± 0.014%	0.028% ± 0.036%	0.087% ± 0.070%	33.06%	0.0016
	*Muriventricola*	1.326% ± 0.404%	3.049% ± 0.906%	1.756% ± 0.607%	1.988% ± 0.553%	−41.24%	< 0.001
Unclear	*Lawsonibacter*	3.369% ± 4.006%	2.644% ± 1.195%	1.543% ± 0.481%	1.938% ± 0.739%	−23.92%	0.0126

Abbreviations: ND, normal diet; WD, western diet; WD‐T, western diet + Totum‐448 (3.5% or 5%).

### 3.2 On/off Study (18 Weeks)

#### 3.2.1 Energy Intake, Body Weight, and Body Composition

**FIGURE 5:**
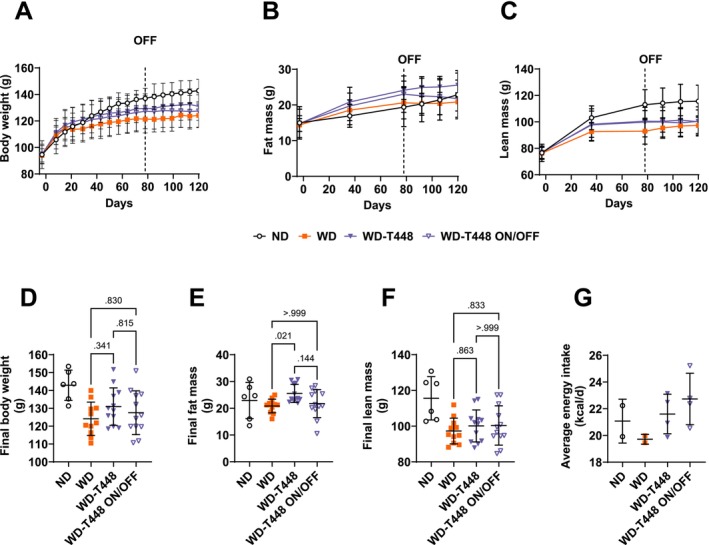
Measures of body weight, body composition, and energy intake in the on/off study: No significant effect of Totum‐448 supplementation or its interruption was observed in body weight or body composition in WD‐fed hamsters. (A) Body weight measured weekly. (B) Fat mass measured on Day ‐3, 36, 78, 92, 106, and 120. (C) Lean mass measured on Day ‐3, 36, 78, 92, 106, and 120. For the sake of readability of the figure, statistical tests were carried on final points only (D–F). (D) Final body weight (Day 120, Week 18). (E) Final fat mass (day 120, week 18). (F) Final lean mass (Day 120, Week 18). *N* = 6–12 animals. (G) Energy intake in each cage, averaged by animal. *N* = 2–4 cages (3 animals per cage). The measurements from two cages in group WD were taken out due to spillage. Because of the low number of replicates, no statistical tests were carried out on energy intake. Post hoc comparisons are displayed on the graph if ANOVA (one‐way or nonparametric equivalent) was significant (*p* < 0.05). The “OFF” mention on the graphs indicates interruption of supplementation in group WD‐T448 ON/OFF.

Like in the 12‐week study, the WD group appeared to display lower body weight compared to ND (Final body weight: 124.1 ± 9.3 vs. 142.9 ± 8.5 g, Figure 5A). No significant differences were observed in body weight between groups WD, WD‐T448, and WD‐T448 ON/OFF (WD: 124.1 ± 9.3 vs. WD‐T448: 130.9 ± 10.5 g, *p* = 0.341, and vs. WD‐T448 ON/OFF: 127.5 ± 12.3, *p* = 0.815, Figure 5A,D). Fat mass and lean mass over time are presented in Figure 5B,C. At the end of the study (Week 18) fat mass was increased in WD‐T448 versus WD (25.56 ± 3.39 vs. 20.86 ± 2.57 g, *p* = 0.021, Figure 5E). Fat mass decreased after interruption of supplementation for 6 weeks in group WD‐T448 ON/OFF, although the difference compared to WD‐T448 was not significant (21.76 ± 5.22 vs. 25.56 ± 3.39, *p* = 0.144, Figure 5B,E). Final (Week 18) lean mass was not different among groups (Figure 5F). Average energy intake over the study period is presented in Figure 5G. Due to significant spillage, measurements were excluded for 2 out of 4 cages in group WD. Energy intake may have been higher in Totum‐448‐supplemented animals, although the exclusion of 50% of the data in group WD calls for caution when interpreting this result (ND: 21.1 ± 1.6, WD: 19.7 ± 0.4, WD‐T448: 21.6 ± 1.5, and WD‐T448 ON/OFF: 22.7 ± 1.9 kcal/days, Figure 5G).

#### 3.2.2 Serum Lipids and Transaminases

**FIGURE 6:**
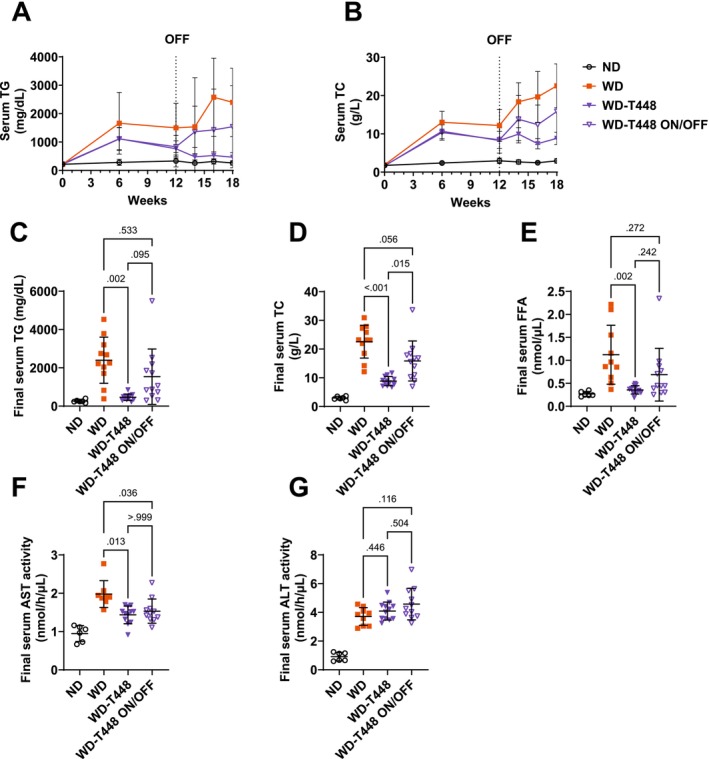
Serum lipids and transaminases in the on/off study: In WD‐fed hamsters, the interruption of Totum‐448 supplementation elicited a progressive increase in serum TG and TC without reaching, after 6 weeks of supplementation cessation, the levels of group WD. The benefits in serum AST seemed to persist despite interruption of supplementation. Serum TG (A) and serum TC (B) were measured at Week 1, Week 6, Week 12, Week 14, Week 16, and Week 18 from blood collected from the gum vein. For the sake of readability of the figure, statistical tests were run on the final point only (Figure C and D). (C) Final (Week 18) serum TG. (D) Final (Week 18) serum TC. (E) Final (Week 18) serum FFA from blood collected from cardiac puncture in anesthetized animals. (F) Final (Week 18) AST activity. (G) Final (Week 18) ALT activity. *N* =6–12 animals. Post hoc comparisons are displayed on the graph if ANOVA (one‐way or repeated‐measures two‐way, or nonparametric equivalent) was significant (*p* < 0.05). ALT, alanine transaminase; AST, aspartate transaminase; FFA, free fatty acids; TC, total cholesterol; TG, triglycerides. The “OFF” mention on the graphs indicates interruption of supplementation in group WD‐T448 ON/OFF.

Serum TG and TC were measured in serum before (Week 1–12) and after (Week 13–18) interruption of supplementation in group WD‐T448 ON/OFF (Figure 6A,B). Serum TC and TG concentration kept increasing over the study period in group WD. Totum‐448 supplementation in group WD‐T448 reduced serum TG and TC from Week 12 and on, compared to WD. Interruption of supplementation from Week 12 in group WD‐T448 ON/OFF resulted in a visible progressive increase in both circulating lipids. At the end of the study (week 18), animals from group WD‐T448 displayed significantly lower serum TG and TC compared to WD (463.5 ± 47.7 vs. 2397.8 ± 363.5 mg/dL, *p* = 0.002 and 8.84 ± 0.46 vs. 22.57 ± 1.73 g/L, *p* < 0.001, respectively, Figure 6C,D). Group WD‐T448 ON/OFF exhibited an intermediate profile, in‐between WD and WD‐T448 for serum TG (1536.9 ± 417.9 vs. 2397.8 ± 363.5, *p* = 0.533 and vs. 463.5 ± 47.7 mg/dL, *p* = 0.095, respectively, Figure 6C) and serum TC (15.86 ± 2.01 vs. 22.57 ± 1.73, *p* = 0.056 and vs. 8.84 ± 0.46 g/L, *p* = 0.015, respectively, Figure 6D). Similarly, serum FFA levels assessed at the end of the study (week 18) showed a similar pattern (although not statistically significant), with group WD‐T448 ON/OFF situated midway between groups WD and WD‐T448 (0.69 ± 0.17 vs. 1.12 ± 0.20, *p* = 0.272 and vs. 0.36 ± 0.03 nmol/μL, *p* = 0.242, respectively, Figure 6E), while they were significantly reduced in WD‐T448 compared to WD (*p* = 0.02). Finally, hamsters in group WD‐T448 featured significantly lower AST activity compared to WD (1.43 ± 0.07 vs. 1.98 ± 0.12, *p* = 0.013, Figure 6F). Interruption of supplementation did not seem to blunt this effect, as AST activity remained significantly lower in group WD‐T448 ON/OFF versus WD and close to WD‐T448 (1.53 ± 0.10 vs. 1.98 ± 0.12, *p* = 0.036 and vs. 1.43 ± 0.07, *p* > 0.999, respectively, Figure 6F). On the other hand, no significant differences were found between groups as for serum ALT activity (Figure 6G).

#### 3.2.3 Liver Parameters

**FIGURE 7:**
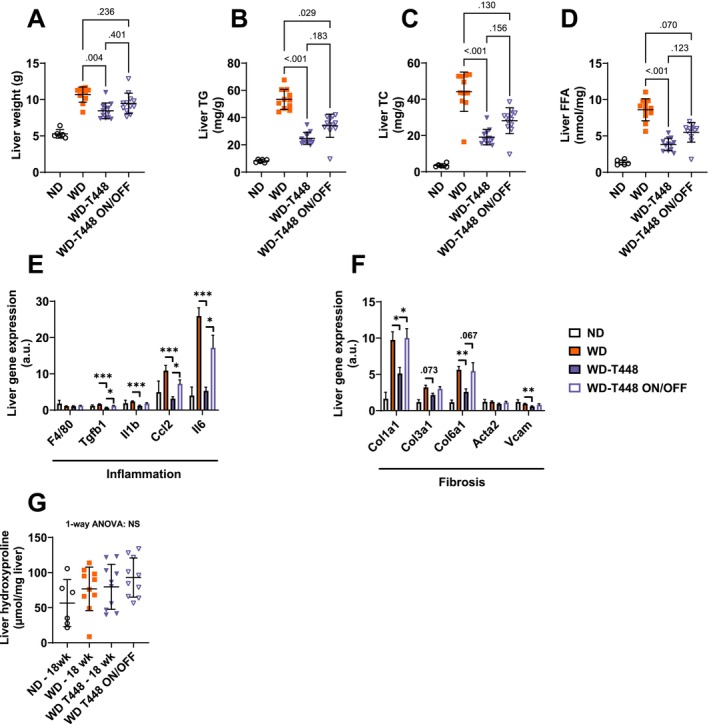
Liver parameters in the on/off study: In WD‐fed hamsters, the interruption of Totum‐448 supplementation resulted in an increase in liver weight and liver lipids, with some benefits preserved, compared to group WD. On the other hand, Totum‐448 effects on gene markers of liver inflammation and fibrosis were totally abolished following supplementation cessation. (A) Liver weight. (B) Liver TG, in mg per gram of wet tissue. (C) Liver TC, in mg per gram of wet tissue. (D) Liver FFA, in nmol per milligram of wet tissue. (E) Relative expression of genes associated with inflammation. (F) Relative expression of genes associated with fibrosis. (G) Liver hydroxyproline expressed in μmol per milligram of wet tissue. *N* =6–12 animals. Post hoc comparisons are displayed on the graph if ANOVA (one‐way or nonparametric equivalent) was significant (*p* < 0.05). *p < 0.05, **p < 0.01, ***p < 0.001. FFA, free fatty acids; TC, total cholesterol; TG, triglycerides.

**FIGURE 8:**
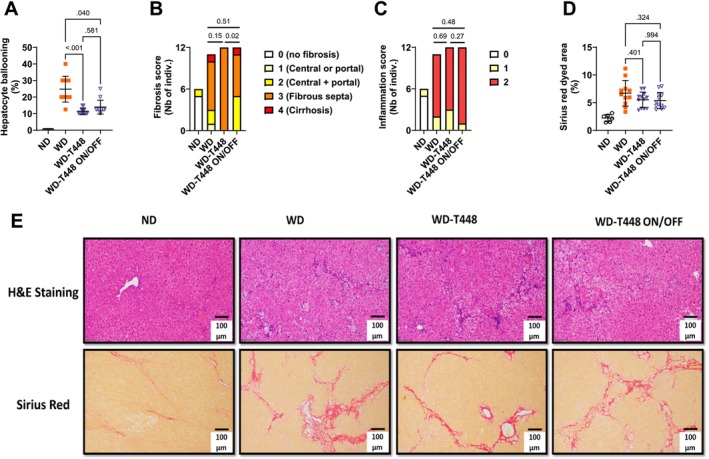
Liver histology in the on/off study: In WD‐fed hamsters, beneficial effects of Totum‐448 were observed in hepatocyte ballooning and persisted despite the interruption of supplementation. However, no benefits were observed in fibrosis or inflammation, as assessed by histological scoring and Sirius red‐dyed area (A–E). Hepatocyte ballooning (A). Fibrosis scoring (B). Inflammation scoring (C). Sirius red‐dyed area (D). Representative images of H&E and Sirius red‐stained liver sections (E). *N* = 6–12 animals. (A, D) Post hoc comparisons are displayed on the graph if ANOVA (one‐way or nonparametric equivalent) was significant (*p* < 0.05). (B, C) *p* values indicate pairwise *χ* ^2^ tests. H&E, hematoxylin and eosin.

Liver weight was decreased in Totum‐448‐supplemented animals in group WD‐T448, compared to WD (8.45 ± 0.31 vs. 10.69 ± 0.31 g, *p* = 0.004, Figure 7A). Liver weight in group WD‐T448 ON/OFF was found in between these two groups, without any statistically significant differences with any of them. Liver lipids were significantly decreased in WD‐T448 compared to WD (TG: 24.80 ± 1.27 vs. 53.38 ± 2.22 mg/g liver, *p* < 0.001, Figure 7B; TC: 19.11 ± 1.23 vs. 44.17 ± 3.27 mg/g liver, *p* < 0.001, Figure 7C; FFA: 3.84 ± 0.25 vs. 8.60 ± 0.46 nmol/mg liver, *p* < 0.001, Figure 7D). Here again, hamsters in group WD‐T448 ON/OFF displayed an intermediate profile following interruption of supplementation, with all liver lipids rising but remaining lower than group WD (TG: 34.03 ± 2.46 vs. 53.38 ± 2.22 mg/g liver, *p* = 0.029, Figure 7B; TC: 28.18 ± 2.07 vs. 44.17 ± 3.27 mg/g liver, *p* = 0.13, Figure 7C; FFA: 5.49 ± 0.39 vs. 8.60 ± 0.46 nmol/mg liver, *p* = 0.07, Figure 7D respectively). Additionally, four gene markers of inflammation (*Il1b*, *Ccl2*, *Tgfb1*, *Il6*, Figure 7E) and 4 gene markers of fibrosis (*Col1a1*, *Col3a1* (trend only, *p* = 0.073), *Col6a1*, *Vcam*, Figure 7F) were significantly downregulated in group WD‐T448 compared to WD. Interruption of supplementation in group WD‐T448 ON/OFF resulted in an increase in the expression of these genes to a level similar to that of group WD (Figure 7E,F). Elevated hydroxyproline levels (a marker of collagen content) were found in all WD groups, compared to ND, without any significant effect of Totum‐448 supplementation (Figure 7G). Finally, histological analyses of liver sections revealed a significant effect of Totum‐448 in reducing hepatocyte ballooning, compared to WD (11.46% ± 0.57% vs. 24.77% ± 2.35%, *p* > 0.001; Figure 8A), a beneficial effect that was conserved despite interruption of supplementation in group WD‐T448 ON/OFF, versus WD (13.96% ± 1.21% vs. 24.77% ± 2.35%, *p* = 0.04). Fibrosis scoring did not show any significant improvement of Totum‐448 compared to the WD group (*p* = 0.15, Figure 8B) but the interruption of supplementation in group WD‐T448 ON/OFF resulted in a significant increase of this score (*p* = 0.02, Figure 8B). No differences among groups were noted for histological inflammation (Figure 8C) or Sirius red‐colored area fraction (Figure 8D). Representative images of H&E and Sirius Red‐stained sections are shown in Figure 8E.

#### 3.2.4 Cecum Microbiota

##### 3.2.4.1 Average Relative Composition

Average relative composition at phylum, class, order, family, and genus level is presented in Figure S4 (A, B, C, D, and E, respectively). Differences between ND and WD are visible. Like in the dose–response study, although no statistical analysis was carried out, Totum‐448 seemed to exacerbate the existing differences between WD and ND, with the notable exception of the family level (Figure S4D), where Totum‐448 tended to restore the relative abundance profile toward that of group ND. Interruption of supplementation in group WD‐T448 ON/OFF resulted in the loss of the effect at all levels.

##### 3.2.4.2 Alpha and Beta‐Diversity

Alpha diversity was evaluated using the Chao and Shannon indexes across the five previously mentioned levels. No differences between groups were observed in the Chao index, except at the class level (Figure S5B), where the index was reduced in group WD, restored in group WD‐T448, and exhibited an intermediate profile in group WD‐T448 ON/OFF. Significant differences between groups were identified in the Shannon index at the family and genus levels (Figure S5I,J). There was a trend toward an increased Shannon index in group WD (particularly at the genus level), which was further amplified in group WD‐T448, while group WD‐T448 ON/OFF displayed an intermediate profile.

Beta diversity assessed by Jaccard and Bray–Curtis indexes at all five levels showed significant group effects at all levels for both indexes (except Jaccard index at order level, Figure S6). Groups ND and WD appeared clearly distinct with both indexes, however, Totum‐448 supplementation did not seem to reduce the distance to group ND, as group WD‐T448 was separate from both ND and WD groups. No clear pattern was identifiable for group WD‐T488 ON/OFF (towards WD or WD‐T448), as it remained close to WD‐T448 with Jaccard index while it seemed to split between ND and WD with Bray–Curtis index.

##### 3.2.4.3 Indicator Taxa

**FIGURE 9:**
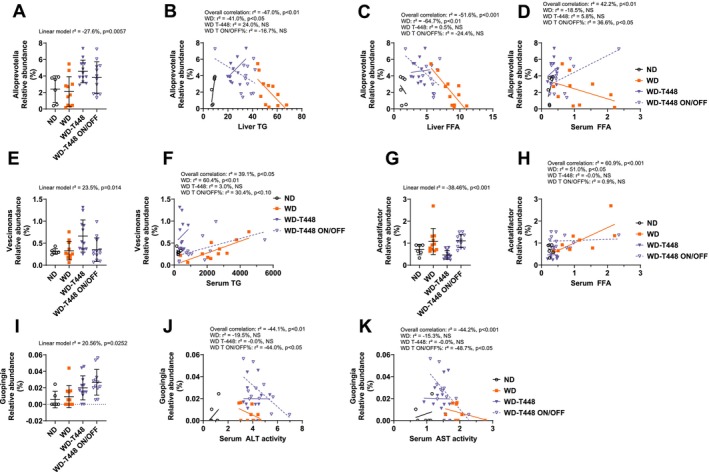
Analysis of cecal microbiota at the genus level in the on/off study and correlations with metabolic outcomes: The relative abundance of 4 taxa at the genus level that presented a significant group effect was significantly correlated with several metabolic outcomes associated with MASLD in WD‐fed hamster. Correlations between indicator bacterial taxa and biological parameters were assessed through linear models whenever both variables displayed a significant difference between groups. (A) *Alloprevotella* relative abundance. (B) Significant linear correlation between *Alloprevotella* relative abundance and liver TG. (C) Significant linear correlation between *Alloprevotella* relative abundance and liver FFA. (D) Significant linear correlation between *Alloprevotella* relative abundance and serum FFA. (E) *Vescimonas* relative abundance. (F) Significant linear correlation between *Vescimonas* relative abundance and serum TG. (G) *Acetatifactor* relative abundance. (H) Significant linear correlation between *Acetatifactor* relative abundance and serum FFA. (I) *Guopingia* relative abundance. (J) Significant linear correlation between *Guopingia* relative abundance and serum ALT activity. (K) Significant linear correlation between *Guopingia* relative abundance and serum AST activity. *N* =6–122 animals. Linear models. *r* ^2^ is the square sum of variances explained by the model, divided by the total variance, (in %). A negative sign was added to indicate a negative correlation. ALT, alanine transaminase; AST, aspartate transaminase; FFA, free fatty acids; TG, triglycerides.

Table 4 shows all the genera with a significant group effect on their relative abundance (unclassified genera with significant differences are presented in Table S3). Like in the dose–response study, we classified them depending on their pattern. Six bacteria were affected by Totum‐448 “beyond WD”: *Paramuribaculum*, *Guopingia*, *Paraprevotella*, *Mucispirillum*, *Acutalibacter*, and *Dorea*. Interruption of supplementation in group WD‐T448 ON/OFF resulted in the loss of Totum‐448 effects for *Paraprevotella*, *Mucispirillum*, *Acutalibacter*, and *Dorea*. Ten genera were categorized in a “T‐448‐specific” pattern: *Rumonococcoides*, *Rutheribacterium*, *Anaerotignum*, *Alloprevotella*, *Vescimonas*, *Pseudoflavonifractor*, *Duncaniella*, *Adlercreutzia*, *Schaedlerella*, and *Neglecta*. The effect was loss in group WD‐T448 ON/OFF for *Vescimonas*, *Pseudoflavonifractor*, *Duncaniella*, *Adlercreutzia*, *Schaedlerella*, and *Neglecta*. Finally, 12 genera displayed a “Back towards ND” pattern: *Muribaculum*, *Intestinimonas*, *Olsenella*, *Bifidobacterium*, *Allobaculum*, *Turicibacter*, *Lactobacillus*, *Muriventricola*, *Acetatifactor*, *Blautia*, *Ihubacter*, and *Dysosmobacter*. The effect was lost in group WD‐T448 ON/OFF for *Turicibacter*, *Lactobacillus*, *Muriventricola*, *Acetatifactor*, *Blautia*, *Ihubacter*, and *Dysosmobacter*. Finally, we found that four taxa presented a significant correlation with a metabolic parameter assessed in these animals. *Alloprevotella* relative abundance (Figure 9A) was negatively associated with liver TG and liver FFA (respectively: *r* ^2^ = −0.470, *p* < 0.01, Figure 9B and *r* ^2^ = −0.516, *p* < 0.001, Figure 9C) and serum FFA, although the latter correlation was not as clear and seemed to be driven by group WD only (Figure 9D). *Vescimonas* relative abundance (Figure 9E) was positively associated with serum TG (*r* ^2^ = 0.391, *p* < 0.01, Figure 9F), *Acetatifactor* relative abundance (Figure 9G) was associated with increased serum FFA (*r* ^2^ = 0.609, *p* < 0.001, Figure 9H), and *Guopingia* relative abundance (Figure 9I) negatively correlated with serum ALT (*r* ^2^ = −0.441, *p* < 0.01, Figure 9J) and serum AST (*r* ^2^ = −0.442, *p* < 0.001, Figure 9K). Additionally, two other significant interactions were identified, exhibiting differential patterns across groups, which complicates their interpretation. *Adlercreutzia* relative abundance (Figure S7A) was increased in group WD‐T448 but negatively correlated in this group with liver TC, while it was decreased in group WD and positively correlated with this parameter (Figure S7B) and *Intestinimonas* (Figure S7C) relative abundance was decreased in group Totum‐448 but positively correlated and serum FFA, while it negatively correlated with this outcome in group WD (Figure S7D).

**TABLE 4:** Analysis of cecal microbiota of hamsters in the on/off study: Significant changes in relative abundance of classified taxa at the genus level.

Effect of T‐448	Effect of interruption (OFF)	Name	Mean ± SD				*r* ^2^	*p*
			ND	WD	WD T‐448	WD T‐448 ON/OFF		
Beyond WD	Effects conserved	*Paramuribaculum*	0.885% ± 0.445%	0.615% ± 0.544%	0.240% ± 0.086%	0.141% ± 0.152%	−30.14%	0.0032
		*Guopingia*	0.006% ± 0.010%	0.009% ± 0.013%	0.020% ± 0.015%	0.027% ± 0.016%	20.56%	0.0252
	Effects lost	*Paraprevotella*	1.296% ± 0.806%	1.791% ± 1.102%	4.497% ± 1.881%	1.307% ± 0.676%	−55.66%	< 0.001
		*Mucispirillum*	0.182% ± 0.069%	0.366% ± 0.187%	0.557% ± 0.304%	0.184% ± 0.161%	−33.56%	0.0014
		*Acutalibacter*	0.070% ± 0.028%	0.045% ± 0.037%	0.006% ± 0.006%	0.119% ± 0.129%	−28.74%	0.0044
		*Dorea*	0.090% ± 0.041%	0.121% ± 0.052%	0.429% ± 0.177%	0.242% ± 0.111%	52.35%	< 0.001
T‐448 specific	Effects conserved	*Ruminococcoides*	0.192% ± 0.150%	0.227% ± 0.223%	0.012% ± 0.033%	0.000% ± 0.000%	−41.90%	< 0.001
		*Ruthenibacterium*	0.102% ± 0.018%	0.118% ± 0.046%	0.196% ± 0.078%	0.177% ± 0.079%	19.61%	0.0304
		*Anaerotignum*	0.118% ± 0.057%	0.102% ± 0.045%	0.179% ± 0.09%	0.186% ± 0.065%	23.97%	0.0125
		*Alloprevotella*	2.412% ± 1.612%	2.118% ± 1.802%	4.561% ± 1.363%	3.814% ± 1.918%	27.64%	0.0057
	Effects lost	*Vescimonas*	0.312% ± 0.065%	0.326% ± 0.208%	0.662% ± 0.365%	0.351% ± 0.268%	23.52%	0.0137
		*Pseudoflavonifractor*	0.003% ± 0.004%	0.000% ± 0.002%	0.025% ± 0.028%	0.003% ± 0.006%	32.69%	0.0018
		*Duncaniella*	2.385% ± 2.029%	2.149% ± 1.725%	5.833% ± 2.605%	2.109% ± 2.788%	36.30%	< 0.001
		*Adlercreutzia*	0.004% ± 0.005%	0.005% ± 0.004%	0.023% ± 0.014%	0.008% ± 0.007%	43.56%	< 0.001
		*Schaedlerella*	0.053% ± 0.046%	0.045% ± 0.051%	0.698% ± 0.334%	0.173% ± 0.133%	65.93%	< 0.001
		*Neglecta*	0.096% ± 0.055%	0.057% ± 0.021%	0.496% ± 0.192%	0.122% ± 0.093%	72.32%	< 0.001
Back towards ND	Effects conserved	*Muribaculum*	0.492% ± 0.208%	0.256% ± 0.161%	0.633% ± 0.324%	0.460% ± 0.282%	26.36%	0.0075
		*Intestinimonas*	0.166% ± 0.047%	0.235% ± 0.059%	0.189% ± 0.059%	0.130% ± 0.041%	−40.89%	< 0.001
		*Olsenella*	0.006% ± 0.008%	0.000% ± 0.002%	0.002% ± 0.004%	0.009% ± 0.014%	17.25%	0.0483
		*Bifidobacterium*	0.089% ± 0.036%	0.013% ± 0.026%	0.031% ± 0.034%	0.341% ± 0.518%	21.22%	0.022
		*Allobaculum*	1.534% ± 0.528%	0.050% ± 0.111%	0.101% ± 0.19%	1.204% ± 0.000%	22.11%	0.0184
	Effects lost	*Turicibacter*	0.001% ± 0.002%	0.052% ± 0.096%	0.000% ± 0.000%	0.000% ± 0.000%	18.27%	0.0397
		*Lactobacillus*	0.011% ± 0.018%	0.105% ± 0.185%	0.000% ± 0.000%	0.000% ± 0.000%	19.51%	0.031
		*Muriventricola*	1.526% ± 0.458%	3.335% ± 0.969%	1.737% ± 0.468%	3.132% ± 1.021%	−43.32%	< 0.001
		*Acetatifactor*	0.709% ± 0.239%	1.077% ± 0.593%	0.465% ± 0.199%	1.103% ± 0.300%	−38.46%	< 0.001
		*Blautia*	0.002% ± 0.003%	0.138% ± 0.106%	0.028% ± 0.065%	0.022% ± 0.044%	−34.82%	0.0011
		*Ihubacter*	0.000% ± 0.000%	0.009% ± 0.011%	0.001% ± 0.003%	0.004% ± 0.004%	−21.44%	0.0211
		*Dysosmobacter*	0.820% ± 0.566%	0.539% ± 0.322%	0.914% ± 0.426%	0.529% ± 0.300%	22.11%	0.0183

Abbreviations: ND, normal diet; WD, western diet; WD T‐448, Western diet + Totum‐448 5%.

## 4 Discussion

This study evaluated for the first time the effects of Totum‐448, a polyphenol‐rich blend of 5 plant extracts and choline, on MASLD in WD‐fed hamsters. Our first hypothesis was validated as the dose–response study revealed dose‐dependent beneficial effects after 12 weeks on key pathological features, such as markers of liver steatosis (hepatic TG, TC, and FFA), relative expression of gene markers of liver inflammation, and, to a lesser extent, of hepatic fibrosis (observed only with the highest dose of Totum‐448). These benefits were accompanied by reductions in circulating lipids in Totum‐448‐supplemented groups (serum TC and TG) with no significant difference between the two doses. Importantly, these effects occurred independently of changes in energy intake, body composition, or glucose homeostasis. The findings were further corroborated in the 18‐week on/off study, as most of the beneficial effects of Totum‐448 on key outcomes related to MASLD pathogenesis were consistently observed, regardless of the experimental setups, in the 12‐week dose–response study and the 18‐week on/off study. The reproducibility of these effects across two independent experimental designs underscores the robustness of Totum‐448's benefits on features of MASLD pathogenesis and strengthens the findings of this study. Additionally, a significant effect on liver ballooning and serum AST levels was observed in the 18‐week experiment, whereas no such effect was detected in the 12‐week trial. Although this work was not designed to evaluate the timeline for the onset and resolution of MASLD features, these findings suggest that certain therapeutic effects of Totum‐448 may require a longer duration of supplementation to manifest fully.

The second hypothesis was also validated, as the interruption of Totum‐448 supplementation led to a gradual loss of its beneficial effects, although some improvements were partially retained. Serum TG and TC began to increase as early as 2 weeks after the discontinuation of the supplementation, and by 6 weeks (Week 18), none of the serum lipid parameters (TC, TC, or FFA) showed significant differences compared with the WD group. In the liver, the effects of Totum‐448 in modulating the expression of genes associated with inflammation and fibrosis were entirely abolished. This is consistent with the transient nature of gene expression changes, which reflect an immediate state of activation rather than long‐term structural alterations. Nevertheless, some benefits were preserved in the liver, particularly the reduction in hepatic TG levels, suggesting the existence of some degree of residual protection in liver steatosis. However, this effect is likely to diminish further over time with continued exposure to the WD, eventually leading to a complete loss of benefits.

The analysis of cecal microbiota revealed significant changes in the relative abundance of certain taxa, with 26 affected in the dose–response study and 28 in the on/off study. In the 12‐week dose–response study, the relative abundance of *Lawsonibacter*, *Lactobacillus*, and *Acetatifactor* was reduced in Totum‐448‐supplemented animals compared to WD‐fed controls. Interestingly, all three taxa were positively associated with serum TC. Additionally, *Lactobacillus* was positively correlated with serum TG and liver FFA, while *Acetatifactor* showed a supplemental positive correlation with serum TG. In line with this, in the on/off study, we observed a reduction in *Acetatifactor* relative abundance in Totum‐448‐supplemented hamsters. This effect was abolished after the supplementation was interrupted. Notably, *Acetatifactor* abundance positively correlated with serum FFA, but this association appeared to be driven primarily by the WD group, as groups WD‐T448 and WD‐T448 ON/OFF displayed no significant correlation and showed a flat slope. *Acetatifactor* is increasingly recognized as a pathogenic microbe associated with adverse metabolic and inflammatory outcomes. It has been linked to intestinal inflammation in mice (Lee et al. 2019). In the present study, *Acetatifactor* relative abundance was positively correlated with serum TG and TC levels, consistent with previous findings obtained in high‐fat diet‐fed mice (Zhu et al. 2023). Additional evidence also supports the association of *Acetatifactor* with Type 2 diabetes in mice, in a recent study (Huang et al. 2024). Interestingly, the authors showed that supplementation with polyphenols from the dicaffeoylquinic acids class (also present in Totum‐448) reduced *Acetatifactor* relative abundance and exerted antidiabetic effects. Similarly, another publication in high‐fat diet‐fed mice demonstrated a decrease in *Acetatifactor* abundance following lipid‐lowering treatment with fenofibrate, a pharmacological agent commonly used to manage hyperlipidemia (Wang et al. 2022). Our results regarding the other two genera mentioned hereabove are less consistent with the literature, as *Lawsonibacter* genus is generally considered a positive indicator in humans, associated with butyrate production (Sakamoto et al. 2018) and known to be increased in coffee drinkers, presumably through the action of polyphenols present in the beverage, chlorogenic acid (also present in Totum‐448) being one of the most abundant and studied (Asnicar et al. 2021; Manghi et al. 2024). Similarly, supplementation with strains belonging to the *Lactobacillus* genus has been shown to improve body weight, circulating lipids, and liver steatosis in high‐fat diet‐fed mice (Wang et al. 2020) and golden hamsters (Wang et al. 2024), in contradiction to our results. In this study, the increase in *Lactobacillus* observed in the nonsupplemented WD group positively correlated with negative outcomes, such as serum lipids. This apparent discrepancy with previous literature may be explained by several factors, including strain‐specific effects within the *Lactobacillus* genus, context‐dependent microbial interactions, or the possibility that its proliferation in the WD group reflects a protective adaptation, and that it could thus be interpreted as a potential defense mechanism against rising blood lipid levels. In this case, this proliferation would be inhibited in Totum‐448‐supplemented animals (and ND hamsters), which exhibited a more favorable lipid profile. Supporting this, Zhu et al. (2023) reported a positive, though nonsignificant, association between fecal *Lactobacillus* relative abundance and serum TG and TC in mice fed a high‐fat diet. Importantly, in human studies, Xu et al. (2022) demonstrated that higher Lactobacillus genus abundance was associated with overweight and obesity in Western populations (Xu et al. 2022). In addition, a systematic review by Crovesy et al. (2017) showed that while some Lactobacillus species may support weight loss, others are associated with weight gain and adverse metabolic outcomes, in humans (Crovesy et al. 2017). This distinction calls for caution when interpreting correlations between *Lactobacillus* abundance and metabolic parameters, and highlights the need for strain‐level identification and functional characterization in future studies. On the other hand, the relative abundance of *Alloprevotella* increased in Totum‐448‐supplemented animals and showed a tendency to decrease after supplementation was stopped. *Alloprevotella* exhibited interesting negative correlations with liver TG, liver FFA, and, to a lesser extent, serum FFA, though the latter association was less pronounced in groups WD‐T448 and WD‐T448 ON/OFF. The relative abundance of this genus was found to be elevated after short‐term dietary fiber supplementation in humans (Tian et al. 2021), and this taxon was also associated with improvement of MASLD features following semaglutide treatment in diabetic *db/db* mice (Mao et al. 2024). Additionally, we found that *Vescimonas* relative abundance was increased by Totum‐448 supplementation but showed a positive correlation with serum TG, particularly in groups WD and WD‐T448 ON/OFF. The literature regarding this taxon is scarce, with only one study finding a reduction of *Vescimonas* relative abundance following high‐fat diet exposure in mice (Shao et al. 2024), contrary to our results, where it was unaltered in group WD. These contradictory observations make it difficult to identify a role for this taxon, added to the fact that our data would suggest that a general increase in the relative abundance of this indicator was associated with a negative outcome (elevated serum TG), except in the WD‐T448 group where this relationship was not observed. Finally, a negative correlation was identified between *Guopingia* relative abundance and both AST and ALT serum levels. ALT and AST are transaminases released into the bloodstream, particularly in cases of hepatocyte damage. Noteworthily, only AST levels were significantly improved by Totum‐448 supplementation after 18 weeks, and interestingly, this effect persisted despite supplementation interruption in the WD‐T448 ON/OFF group. Similarly, *Guopingia* relative abundance remained elevated in this group compared to WD, aligning with the sustained improvement in AST levels. This genus was only recently described (Liu et al. 2021), and its potential role in MASLD pathogenesis remains unexplored. While its association with AST and ALT levels in this study is intriguing, a direct causal relationship between the relative abundance of this bacterium and the transaminase levels seems unlikely. Indeed, AST and ALT are transaminases secreted into circulation by hepatocytes, erythrocytes, or myocytes in response to cellular damage. A more plausible explanation could involve an interaction where elevated transaminase levels or other circulating markers of tissue damage influence the composition of the intestinal microbiota, including *Guopingia*. Further investigation is needed to unravel this potential relationship.

Although this study was not specifically designed to elucidate the mechanisms of action underlying Totum‐448's effects, several plausible hypotheses can be considered, based on the existing data. The observed reduction in circulating TG and TC with Totum‐448 supplementation, coupled with the rebound in these parameters as early as 2 weeks after supplementation interruption in the WD‐T448 ON/OFF group, might initially suggest an effect on intestinal lipid absorption. However, this hypothesis is contradicted by the absence of significant changes in body weight or fat mass in the 12‐week study, in roughly isocaloric conditions. In fact, fat mass was slightly increased in the Totum‐448 group during the 18‐week study, likely due to the higher energy intake observed in this group. On the other hand, circulating FFA, which originate from the hydrolysis of adipose tissue TG, may provide an alternative explanation. Elevated serum FFA levels, as seen in the WD group, could indicate an enhanced lipolytic state (Karpe et al. 2011). This is particularly relevant to liver steatosis, as circulating FFA are a primary source of hepatic triglyceride accumulation. The liver actively takes up FFA from the bloodstream and converts them into TG for storage. This process is a key driver of hepatic lipid overload in conditions such as MASLD, where elevated circulating FFA, often stemming from increased adipose tissue lipolysis, exacerbate the progression of steatosis (Donnelly et al. 2005). Supporting this connection, genetic disruption of lipolysis has been shown to reduce hepatic TG storage, further highlighting the critical role of circulating FFA in liver lipid metabolism (Zhang et al. 2024). While the contribution of circulating FFA to hepatic TG accumulation is well‐established, it is important to note that in humans, this process is largely driven by insulin resistance. Insulin resistance promotes increased lipolysis in adipose tissue, leading to elevated circulating FFA and their subsequent uptake by the liver (Utzschneider and Kahn 2006). However, the measurements in this study suggest that the WD‐fed hamster model does not replicate this aspect of MASLD pathophysiology, as fasting glycemia and insulinemia in this model remained unaltered by WD, suggesting that significant insulin resistance may not have developed. Moreover, we noted that WD exposure did not induce fat mass gain in hamsters.

Excessive fat accumulation and the development of insulin resistance are key drivers of human MASLD pathogenesis (Guerra et al. 2022). In particular, insulin resistance in AT plays a central role: when AT approaches its limit for lipid storage, an inflammatory response is triggered and its ability to respond to insulin diminishes. As a result, insulin fails to suppress lipolysis, leading to elevated release of FFAs (Jensen 2006) into circulation. Of note, inflammation of AT alone is sufficient to induce FFA release into the circulation and other pathways leading to AT lipolysis independent of insulin have been shown to be dysregulated in a context of obesity (Folestad and Falkevall 2024; Roh and Yoo 2021). These FFAs are then transported to nonadipose organs such as the liver, where they ectopically accumulate as triglycerides, contributing to hepatic steatosis and progression of MASLD (E. Lee et al. 2023). Interestingly, while this hamster model does not recapitulate obesity‐associated insulin resistance, our data indicate a marked increase in circulating FFAs under WD conditions, similar to the human phenotype. This may reflect a lipolytic state of the AT, potentially driven by metabolic inflexibility or limited expandability, even in the absence of insulin resistance. Thus, although the present work was not designed to elucidate this question, this model may still capture certain pathophysiological aspects of MASLD, particularly the contribution of dysfunctional adipose tissue to hepatic lipid overload. Another major limitation inherent to this work is the relatively high doses used, 3.5% and 5% in diet, that represent ~1.2 to ~1.8 g/kg, respectively, for a 120‐g hamster eating 4.3 g of supplemented WD daily. Two clinical trials are currently underway to evaluate the effects of Totum‐448 in humans with MASLD (NCT06047847 and NCT06704321). The dose of Totum‐448 in these studies is 4.3 g/day, that is, ~0.07 g/kg for a 65‐kg adult. Applying the interspecies dose conversion factor of 7.4 for hamsters (Nair and Jacob 2016) this would correspond to an equivalent dose of ~0.5 g/kg in hamsters. Therefore, in comparison, the doses used in the present study are about 2.5–3.5 times higher than the anticipated human equivalent dose. Although such cross‐species dose conversions should be interpreted cautiously, the promising preclinical results reported here will hence require confirmation in clinical settings using the intended human doses. Finally, we acknowledge that, unlike in a pharmacological study, this proof‐of‐concept design does not allow us to attribute specific effects to individual components of Totum‐448, in particular the individual contribution of polyphenols, choline, and fibers. Further studies are needed to explore Totum‐448's mode of action and whether its effects on lipid metabolism and liver steatosis could extend to settings of pronounced insulin resistance.

In conclusion, this study provides robust evidence of the beneficial effects of Totum‐448 on key features of MASLD in WD‐fed hamsters. Totum‐448 consistently reduced hepatic lipid accumulation, improved relative gene expression of markers of inflammation and fibrosis in the liver, and lowered circulating lipid levels across two experimental setups. These effects were achieved independently of changes in body composition or glucose homeostasis and showed a dose‐dependent response. The transient nature of these benefits following the interruption of supplementation suggests that continuous supplementation is required to maintain efficacy. Additionally, the analysis of cecal microbiota revealed that Totum‐448 supplementation significantly modulated the relative abundance of several key taxa, with notable shifts in *Acetatifactor*, *Alloprevotella*, *Lactobacillus*, and *Lawsonibacter* correlating with metabolic improvements.

## Author Contributions

**Vivien Chavanelle:** conceptualization (equal), data curation (equal), formal analysis (equal), investigation (equal), methodology (equal), project administration (equal), visualization (equal), writing – original draft (equal), writing – review and editing (equal). **Marie Vallier:** conceptualization (equal), data curation (equal), formal analysis (equal), methodology (equal), software (equal), visualization (equal). **Yolanda F. Otero:** conceptualization (equal), formal analysis (equal), investigation (equal), project administration (equal), supervision (equal), validation (equal). **Doriane Ripoche:** methodology (equal), software (equal). **Florian Le Joubioux:** conceptualization (equal), data curation (equal), formal analysis (equal), methodology (equal), software (equal), visualization (equal). **Thierry Maugard:** conceptualization (equal), methodology (equal), project administration (equal), supervision (equal), validation (equal). **Gaël Ennequin:** conceptualization (equal), funding acquisition (equal), project administration (equal), resources (equal), validation (equal). **Valérie Hervieu:** data curation (equal), formal analysis (equal), methodology (equal), resources (equal), software (equal). **Sébastien Peltier:** conceptualization (equal), funding acquisition (equal), project administration (equal), resources (equal), supervision (equal), validation (equal). **Pascal Sirvent:** conceptualization (equal), formal analysis (equal), project administration (equal), resources (equal), supervision (equal), validation (equal), writing – original draft (equal), writing – review and editing (equal).

## Conflicts of Interest

V.C. and F.L.J. are Valbiotis employees. M.V., Y.F.O., and D.R. are former Valbiotis employees. T.M. is a member of Valbiotis' scientific board. S.P. is Valbiotis CEO. P.S. is Valbiotis CSO. G.E. and V.H. declare that they have no known competing financial interests or personal relationships that could have appeared to influence the work reported in this paper.

## Supporting information


Data S1.

## Data Availability

The data that support the findings of this study are available from the corresponding author upon reasonable request.
