# Supplementary material for: Totum‐448 Improves MASLD and Modulates Microbiota in Hamsters: Dose–Response Study and Effects of Supplementation Cessation
Source: Food Sci Nutr. 2025 Sep 2;13(9):e70904. doi: 10.1002/fsn3.70904 (PMC12403019; doi:10.1002/fsn3.70904)

Supplementary materials

Supplementary Table 1: Chemical characterization of Totum-448

| **Compound types (sorted by families)** | **Extract content (g/100 g)** |
| --- | --- |
| Choline* | 13.67 |
| Total phenolic compounds | 8.7 |
| Total anthocyanins | 0.536 |
| Monocaffeoylquinic acids |  |
| Chlorogenic acid | 0.517 |
| Cryptochlorogenic acid | 0.324 |
| Neochlorogenic acid | 0.319 |
| Other monocaffeoylquinic acids | 0.115 |
| Dicaffeoylquinic acids |  |
| Cynarine | 0.229 |
| 4,5-Dicaffeoylquinic acid | 0.098 |
| 3,5-Dicaffeoylquinic acid | 0.074 |
| 3,4-Dicaffeoylquinic acid | 0.056 |
| Caffeic acid | 0.008 |
| Oleuropein | 6.223 |
| Oleuropein isomers | 0.757 |
| Ligstroside | 0.131 |
| Luteolin | 0.017 |
| Luteolin-7-O-glucoside | 0.880 |
| Luteolin-7-O-glucuronide | 0.277 |
| Luteolin-4-O-glucoside | 0.083 |
| Apigenin-7-O-glucoside | 0.062 |
| Apigenin-7-O-glucuronide | 0.139 |
| Apigenin-7-O-rutinoside | 0.037 |
| Verbascoside | 0.152 |
| Terpenes and terpenoids |  |
| Oleanolic acid | 0.199 |
| Cynaropicrin | 0.139 |
| Saponins |  |
| Chrysanthellin A | 0.133 |
| Chrysanthellin B | 0.215 |
| Alkaloids |  |
| Piperin | 0.044 |

* in choline chloride equivalent.

Supplementary materials

Suppl. Table 1 shows the chemical characterization of TOTUM-448. Total phenolic compound levels (in gallic acid equivalent) was assessed using the Folin-Ciocalteu colorimetric method (Singleton et Rossi 1965). A more precise characterization of phytochemical compounds was performed by HPLC-UV/Visible/RID-MS using 1260 LC system and 1200 LC system with a 6110 Single Quad MS-ESI detector (Agilent Technologies, Santa Clara, CA, USA) with a C18 Prodigy reversed-phase column (250 mm × 4.6 mm, 5 μm; Phenomenex, USA) and an Atlantis HILIC Silica column (150×4.6 mm, 5 μm, Waters, The Netherlands).

Singleton, V. L., et Joseph A. Rossi. 1965. « Colorimetry of Total Phenolics with Phosphomolybdic-Phosphotungstic Acid Reagents ». *American Journal of Enology and Viticulture* 16 (3): 144‑58. https://doi.org/10.5344/ajev.1965.16.3.144.

Supplementary Table 2: Analysis of cecal microbiota of hamsters in the dose-response study: significant changes in relative abundance of unclassified taxa at the genus level.

|  |  | **Mean +/- SD** | | | | | | | | | | | |  |  |
| --- | --- | --- | --- | --- | --- | --- | --- | --- | --- | --- | --- | --- | --- | --- | --- |
|  | Name | **ND** | | | **WD** | | | **WD T-3.5%** | | | **WD T-5%** | | | R | *p* |
| Beyond WD | uncl.Eggerthellaceae | 0.029% | +/- | 0.013% | 0.049% | +/- | 0.023% | 0.144% | +/- | 0.069% | 0.123% | +/- | 0.043% | 43.95% | *<0.001* |
|  | uncl.Eubacteriales | 2.849% | +/- | 0.706% | 2.505% | +/- | 0.711% | 2.203% | +/- | 0.695% | 1.781% | +/- | 0.354% | -19.95% | *0.0284* |
|  | uncl.Clostridia | 0.121% | +/- | 0.032% | 0.187% | +/- | 0.053% | 0.282% | +/- | 0.081% | 0.332% | +/- | 0.093% | 40.07% | *<0.001* |
|  | uncl.Bacillota | 1.274% | +/- | 0.440% | 0.590% | +/- | 0.303% | 0.416% | +/- | 0.325% | 0.172% | +/- | 0.086% | -31.04% | *0.0026* |
|  | uncl.Bacteroidales | 1.053% | +/- | 0.487% | 1.688% | +/- | 0.515% | 2.381% | +/- | 0.930% | 2.771% | +/- | 0.492% | 31.94% | *0.0021* |
|  | uncl.Desulfovibrionaceae | 6.227% | +/- | 2.588% | 11.048% | +/- | 2.370% | 9.710% | +/- | 2.194% | 13.677% | +/- | 1.579% | 39.91% | *<0.001* |
|  | uncl.Bacteria | 3.610% | +/- | 0.858% | 3.979% | +/- | 0.476% | 5.400% | +/- | 1.377% | 5.967% | +/- | 0.691% | 46.46% | *<0.001* |
| T-448 specific | uncl.Coriobacteriia | 0.003% | +/- | 0.004% | 0.004% | +/- | 0.006% | 0.020% | +/- | 0.018% | 0.007% | +/- | 0.007% | 28.90% | *0.0043* |
|  | uncl.Oscillospiraceae | 9.141% | +/- | 3.362% | 7.973% | +/- | 1.935% | 6.093% | +/- | 0.732% | 6.024% | +/- | 0.927% | -34.16% | *0.0012* |
| Back to ND | uncl.Lactobacillaceae | 0.027% | +/- | 0.025% | 0.059% | +/- | 0.058% | 0.007% | +/- | 0.021% | 0.003% | +/- | 0.011% | -35.24% | *<0.001* |
|  | uncl.Muribaculaceae | 2.345% | +/- | 0.870% | 0.945% | +/- | 0.319% | 1.450% | +/- | 0.607% | 1.792% | +/- | 0.574% | 33.11% | *0.0016* |
|  | uncl.Bacteroidota | 0.041% | +/- | 0.029% | 0.115% | +/- | 0.055% | 0.065% | +/- | 0.040% | 0.049% | +/- | 0.019% | -33.69% | *0.0014* |
|  | uncl.Sutterellaceae | 0.001% | +/- | 0.002% | 0.146% | +/- | 0.106% | 0.017% | +/- | 0.052% | 0.001% | +/- | 0.003% | -49.16% | *<0.001* |

Supplementary Table 3: Analysis of cecal microbiota of hamsters in the on/off study: significant changes in relative abundance of unclassified taxa at the genus level.

|  |  |  | **Mean +/- SD** | | | | | | | | | | | |  |  |
| --- | --- | --- | --- | --- | --- | --- | --- | --- | --- | --- | --- | --- | --- | --- | --- | --- |
| Effect of T-448 | Effect of interruption (OFF) | Name | **ND** | | | **WD** | | | **WD T-448** | | | **WD T-448 ON/OFF** | | | R | *p* |
| Beyond ND | Lost | uncl.Clostridia | 0.129% | +/- | 0.075% | 0.227% | +/- | 0.043% | 0.388% | +/- | 0.112% | 0.230% | +/- | 0.112% | 40.47% | *<0.001* |
|  |  | uncl.Bacteroidales | 1.481% | +/- | 0.307% | 2.101% | +/- | 0.532% | 3.341% | +/- | 0.285% | 1.716% | +/- | 0.544% | 71.17% | *<0.001* |
| T448-specific | Lost | uncl.Coriobacteriia | 0.007% | +/- | 0.006% | 0.002% | +/- | 0.004% | 0.013% | +/- | 0.015% | 0.004% | +/- | 0.005% | 20.57% | *0.025* |
|  |  | uncl.Eggerthellaceae | 0.023% | +/- | 0.019% | 0.022% | +/- | 0.014% | 0.149% | +/- | 0.078% | 0.048% | +/- | 0.020% | 59.01% | *<0.001* |
|  |  | uncl.Deltaproteobacteria | 0.007% | +/- | 0.005% | 0.006% | +/- | 0.009% | 0.016% | +/- | 0.014% | 0.005% | +/- | 0.005% | 21.55% | *0.021* |
|  |  | uncl.Prevotellaceae | 0.774% | +/- | 0.381% | 0.755% | +/- | 0.312% | 1.395% | +/- | 0.290% | 0.931% | +/- | 0.296% | 47.06% | *<0.001* |
| Back towards ND | Lost | uncl.Sutterellaceae | 0.002% | +/- | 0.004% | 0.027% | +/- | 0.042% | 0.002% | +/- | 0.004% | 0.080% | +/- | 0.107% | 21.01% | *0.023* |
|  |  | uncl.Bacteria | 4.686% | +/- | 1.221% | 4.138% | +/- | 0.989% | 4.923% | +/- | 1.103% | 3.640% | +/- | 0.680% | 26.19% | *0.008* |
|  |  | uncl.Muribaculaceae | 2.133% | +/- | 0.569% | 1.109% | +/- | 0.321% | 2.762% | +/- | 0.503% | 1.236% | +/- | 0.564% | 73.17% | *<0.001* |
|  |  | uncl.Oscillospiraceae | 6.947% | +/- | 0.419% | 8.130% | +/- | 2.016% | 5.581% | +/- | 0.840% | 7.624% | +/- | 1.763% | -34.05% | *0.001* |
|  | Conserved | uncl.Pseudomonadota | 0.095% | +/- | 0.059% | 0.051% | +/- | 0.019% | 0.094% | +/- | 0.057% | 0.115% | +/- | 0.061% | 23.36% | *0.014* |
|  |  | uncl.Erysipelotrichaceae | 1.509% | +/- | 0.681% | 0.296% | +/- | 0.194% | 0.834% | +/- | 0.507% | 2.196% | +/- | 1.814% | 36.17% | *<0.001* |
| No effect | Back towards ND | uncl.Bacillota | 0.439% | +/- | 0.107% | 0.127% | +/- | 0.057% | 0.109% | +/- | 0.062% | 0.330% | +/- | 0.294% | -25.68% | *0.009* |
|  |  | uncl.Lachnospiraceae | 29.360% | +/- | 6.149% | 11.941% | +/- | 5.793% | 11.259% | +/- | 2.410% | 17.492% | +/- | 7.825% | 20.57% | *0.025* |
|  |  | uncl.Bifidobacteriaceae | 0.020% | +/- | 0.024% | 0.002% | +/- | 0.004% | 0.003% | +/- | 0.006% | 0.056% | +/- | 0.086% | 21.91% | *0.019* |
|  |  | uncl.Eubacteriales | 2.453% | +/- | 0.298% | 1.527% | +/- | 0.362% | 1.444% | +/- | 0.222% | 2.031% | +/- | 0.650% | 26.94% | *0.007* |

Suppl. Fig. 1: Hamster dose response study: Average relative cecal microbiota composition at phylum (A), class (B), order (C), family (D), and genus (E) level.


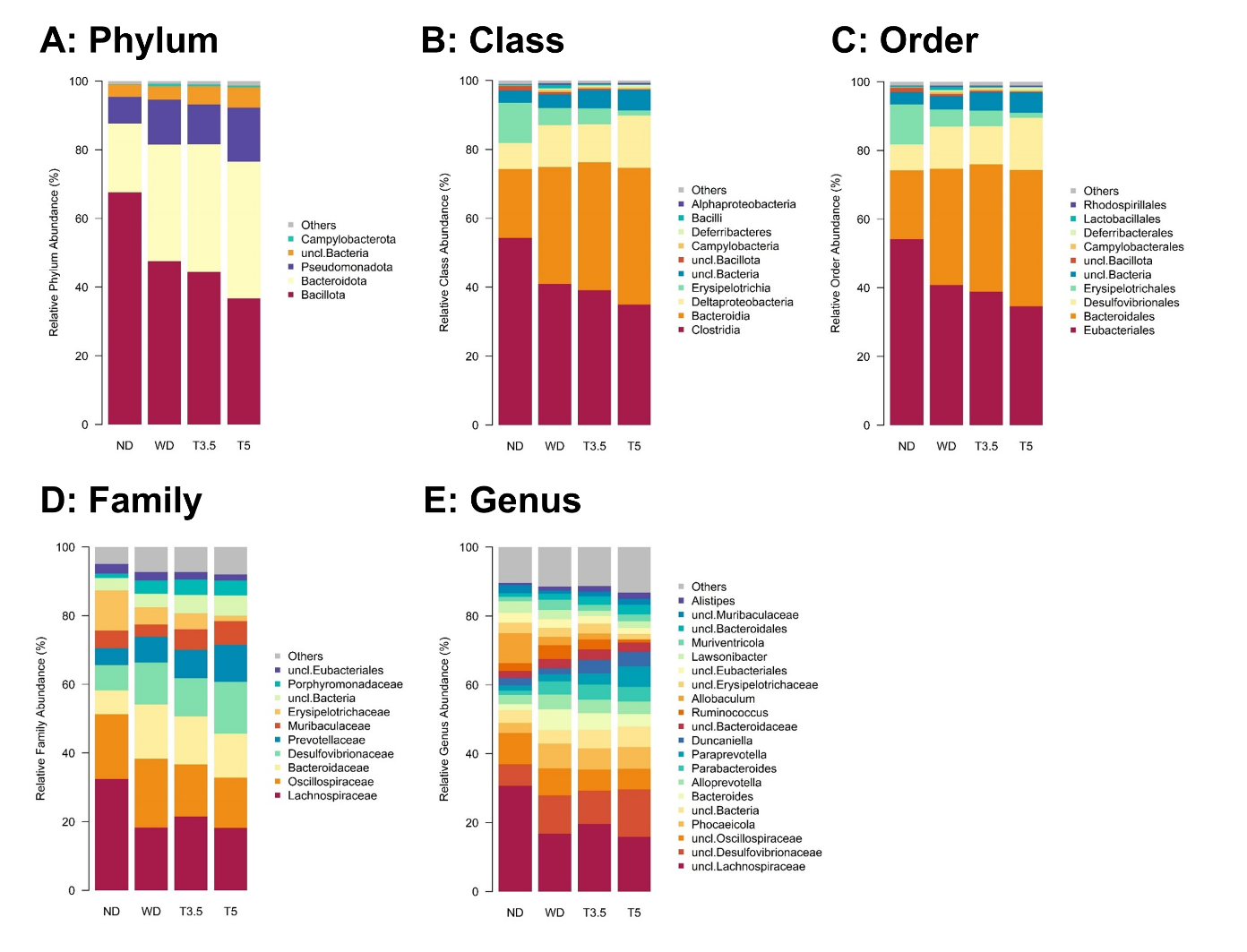


Suppl. Fig. 2: Hamster dose-response study, analysis of cecal microbiota alpha diversity assessed by Chao index at phylum (A), class (B), order (C), family (D), and genus (E) level. Alpha diversity assessed by Shannon index at phylum (F), class (G), order (H), family (I), and genus (J) level. . p<0.10, ** p<0.01, lm R: linear model r²


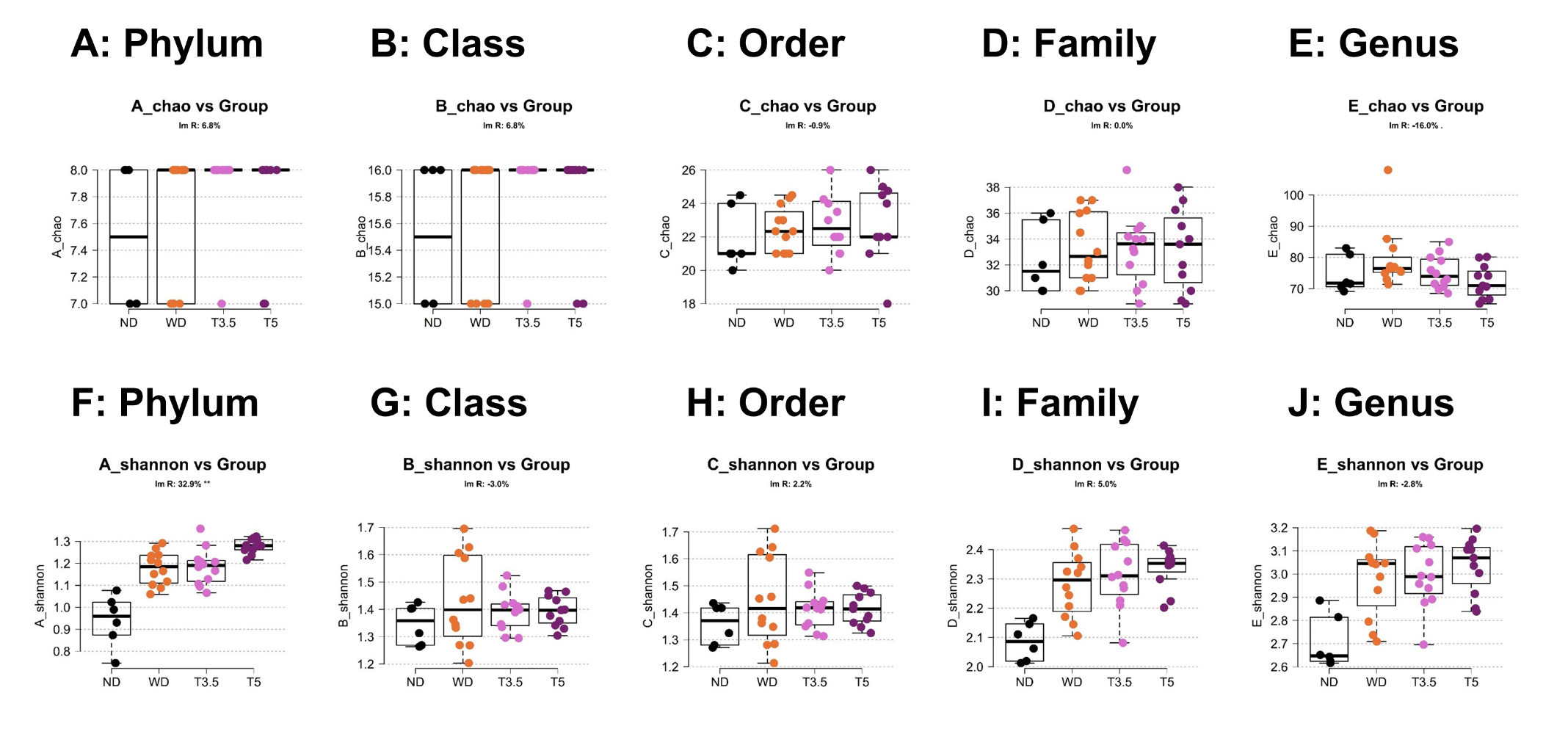


.

Suppl. Fig. 3: Hamster dose response study, analysis of cecal microbiota beta diversity assessed by Jaccard index at phylum (A), class (B), order (C), family (D), and genus (E) level. Beta diversity assessed by Bray-Curtis index at phylum (F), class (G), order (H), family (I), and genus (J) level. . p<0.10, * p<0.05, ** p<0.01, *** p<0.001.


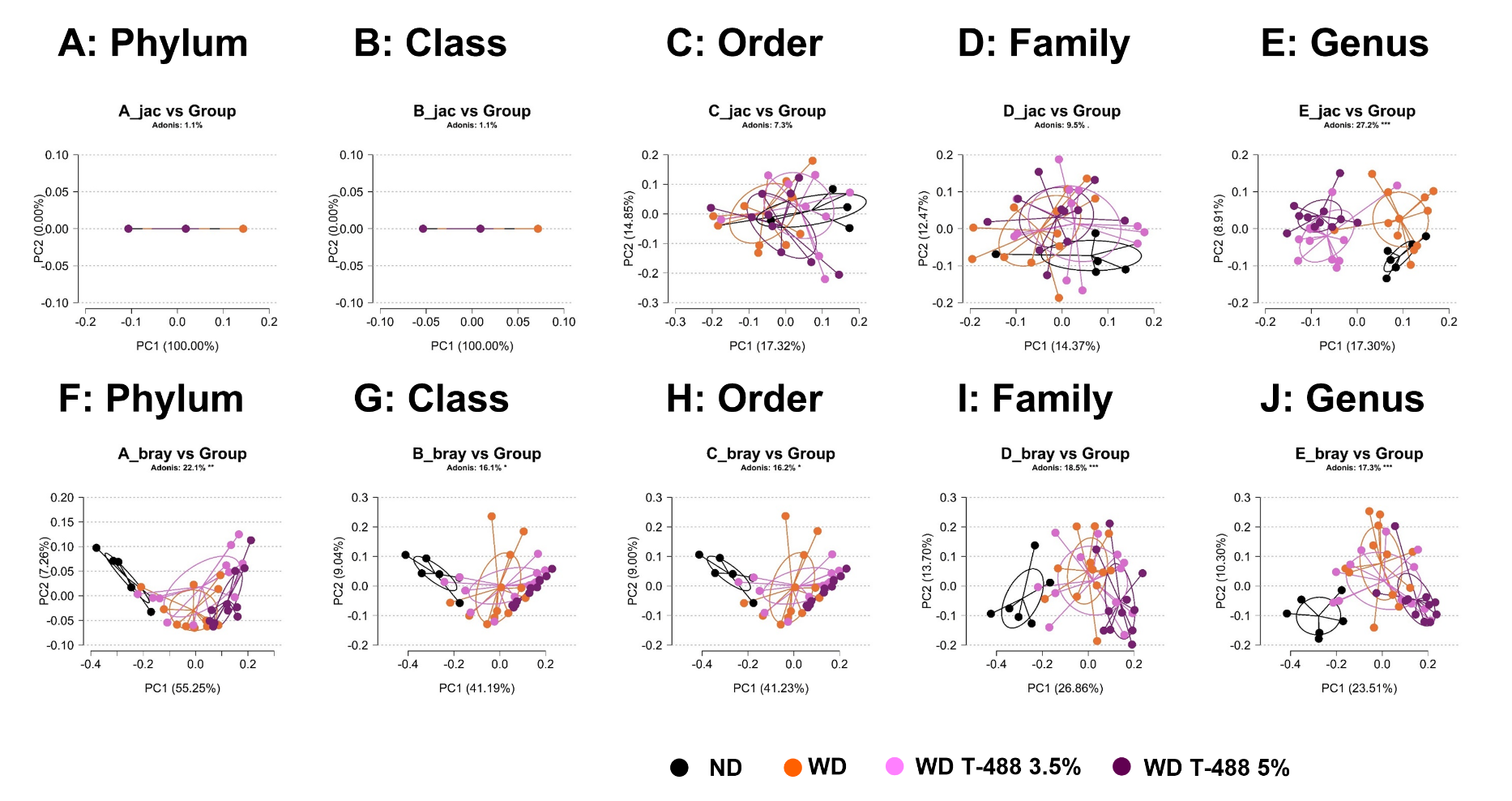


Suppl. Fig. 4: Hamster on/off study, average relative cecal microbiota composition at phylum (A), class (B), order (C), family (D), and genus (E) levels


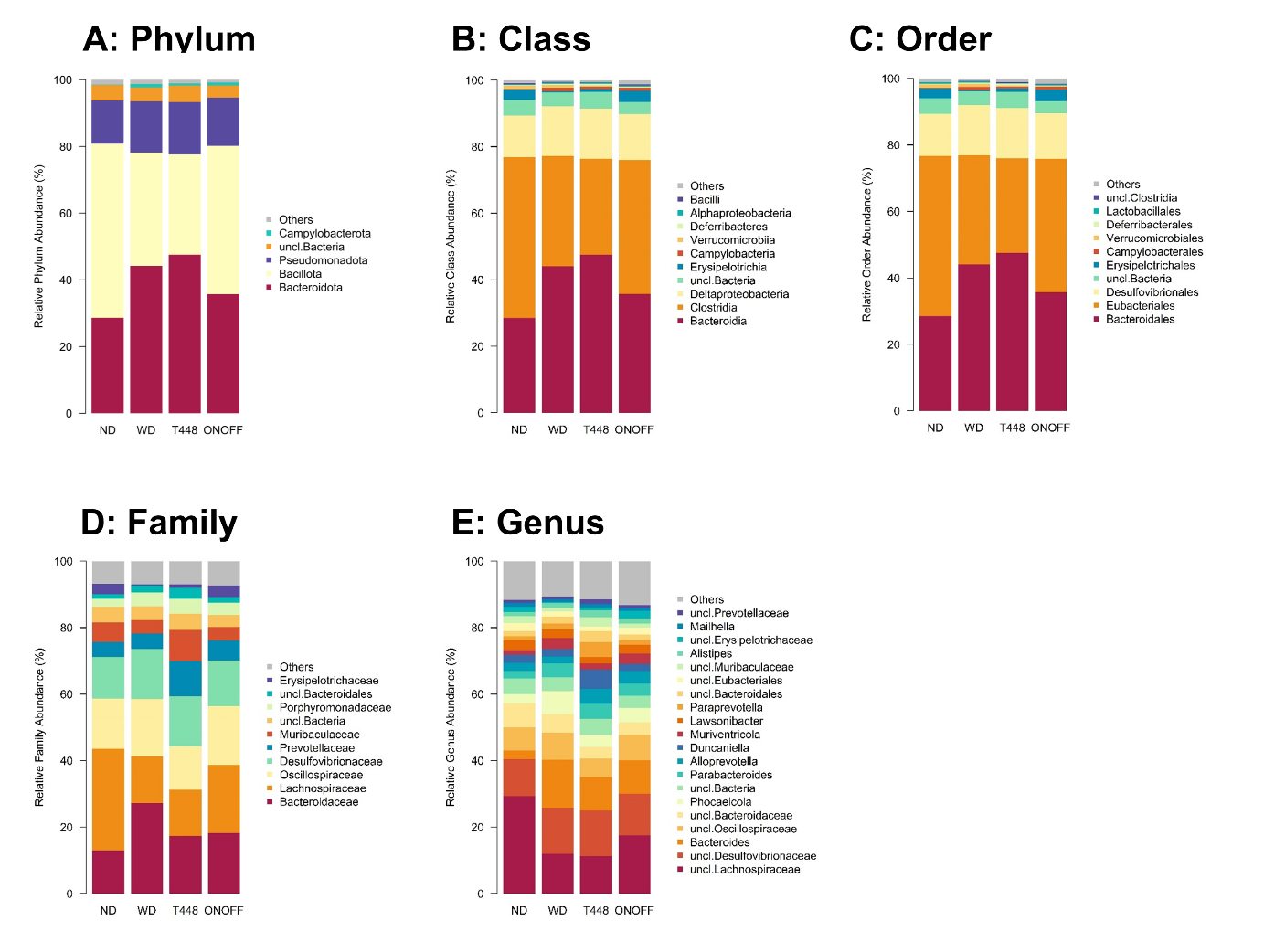


Suppl. Fig. 5: Hamster on/off study, analysis of cecal microbiota alpha diversity assessed by Chao index at phylum (A), class (B), order (C), family (D), and genus (E) level. Alpha diversity assessed by Shannon index at phylum (F), class (G), order (H), family (I), and genus (J) level. *** p<0.01, lm R: linear model r².


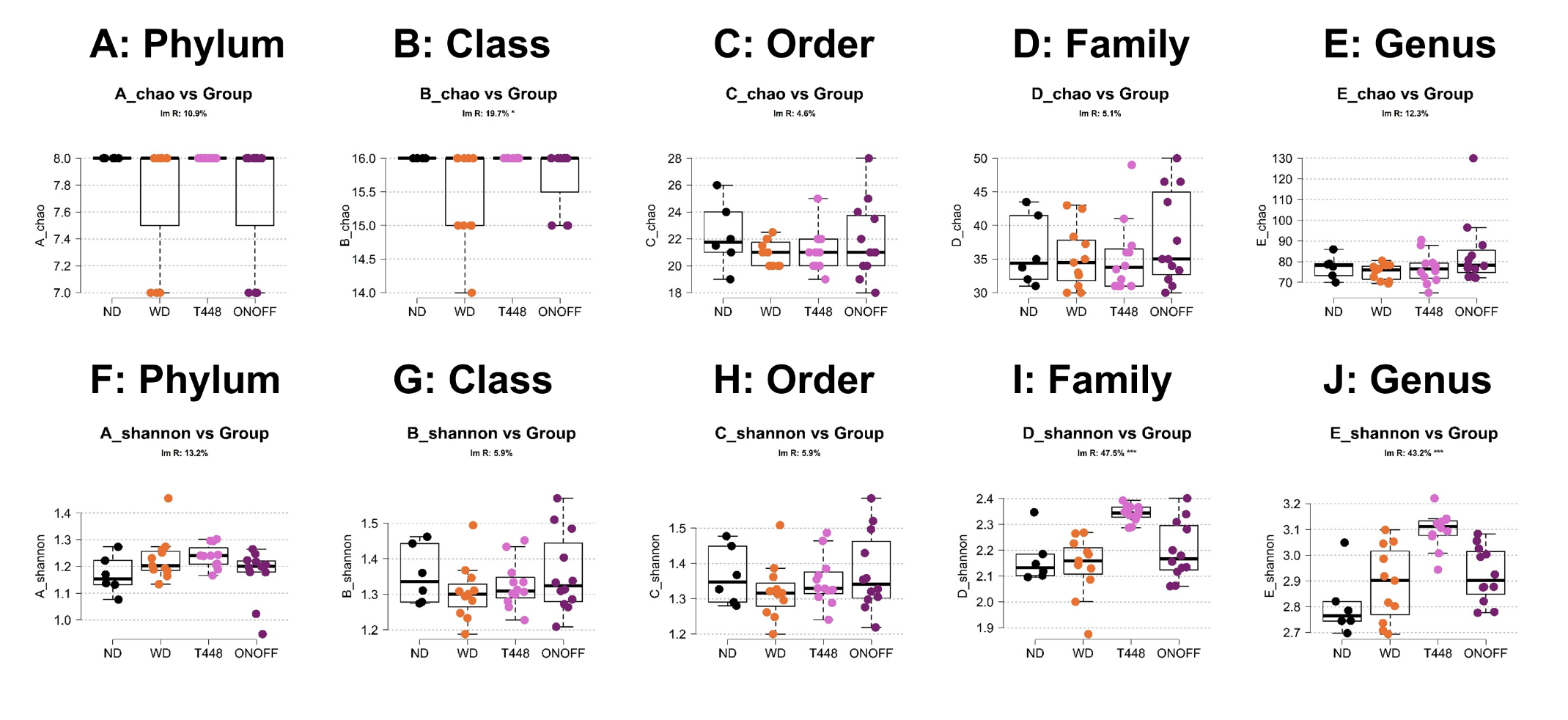


Suppl. Fig. 6: Hamster on/off study, analysis of cecal microbiota beta diversity assessed by Jaccard index at phylum (A), class (B), order (C), family (D), and genus (E) level. Beta diversity assessed by Bray-Curtis index at phylum (F), class (G), order (H), family (I), and genus (J) level. ** p<0.01, *** p<0.001.


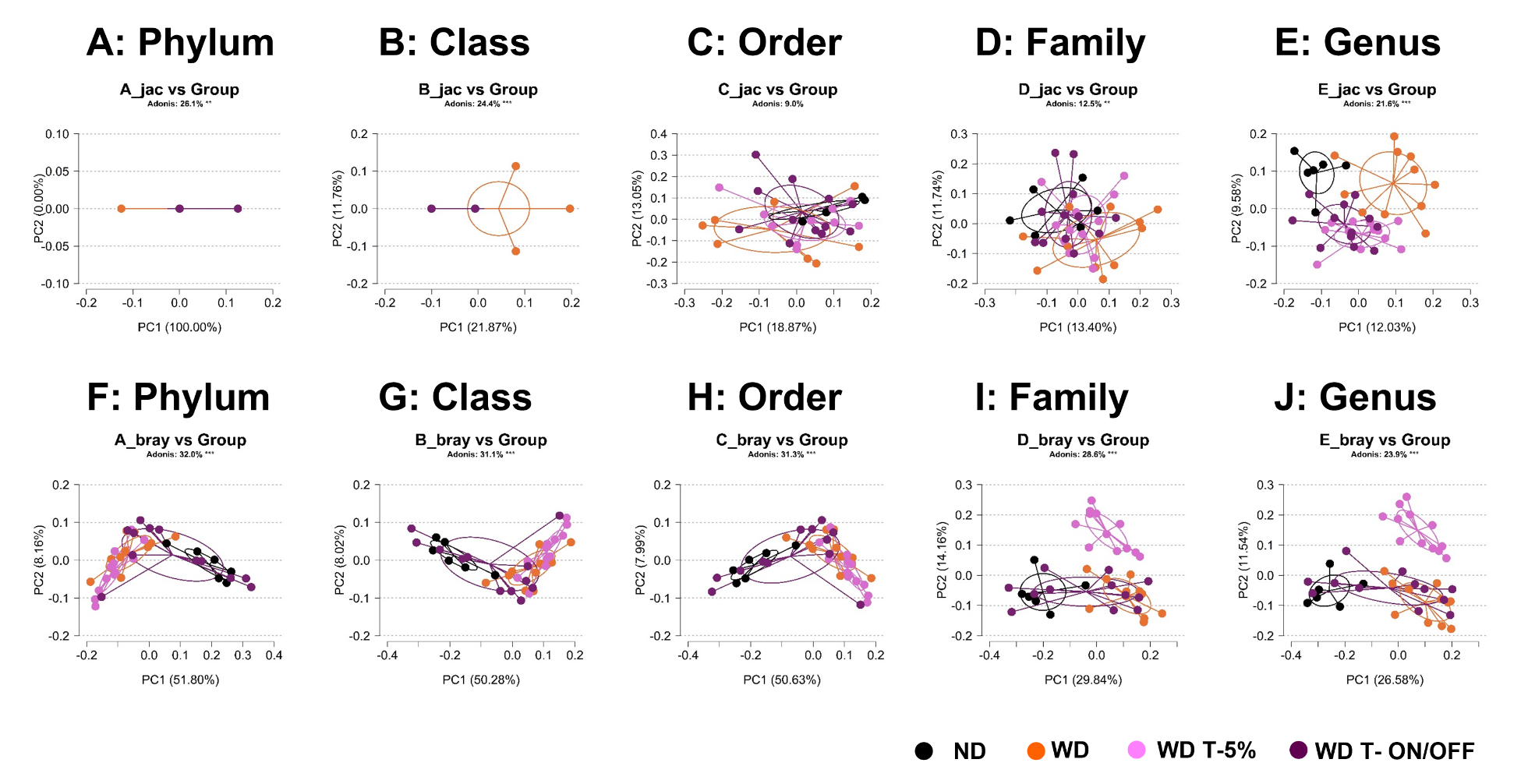


Suppl. Fig. 7: Hamster on/off study, significant correlation between relative abundance of cecal microbiota species at the genus level and a metabolic outcome with unclear interpretation.


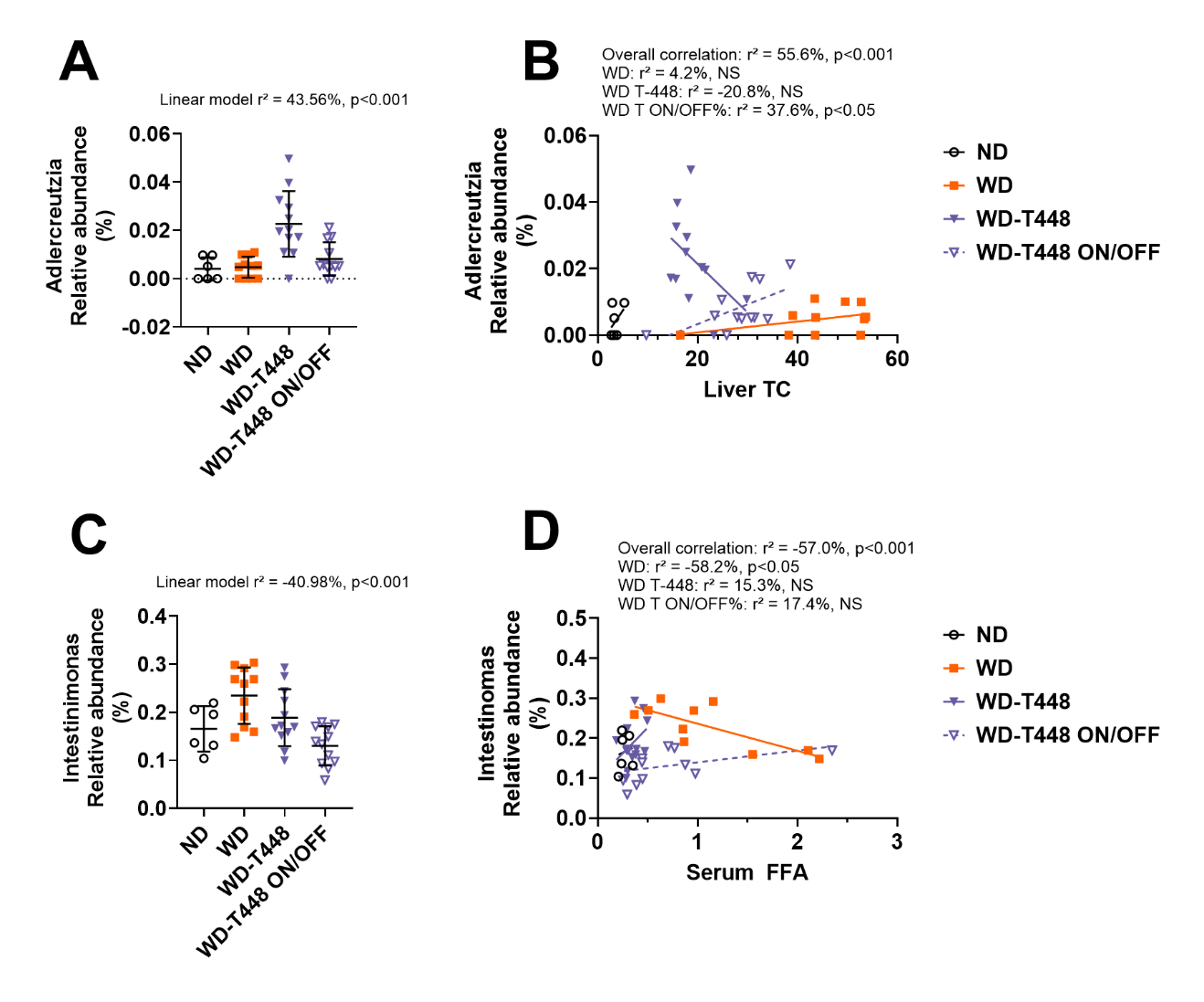

Supplement: Supplementary file 1 — Data S1. [file FSN3-13-e70904-s001.docx]
